# Transcriptional Potential Determines the Adaptability of Escherichia coli Strains with Different Fitness Backgrounds

**DOI:** 10.1128/spectrum.02528-22

**Published:** 2022-11-29

**Authors:** Kitae Kim, Soon-Kyeong Kwon, Pil Kim, Jihyun F. Kim

**Affiliations:** a Department of Systems Biology, Division of Life Sciences, and Institute for Life Science and Biotechnology, Yonsei University, Seoul, Republic of Korea; b Division of Applied Life Science (BK21), Gyeongsang National University, Jinju-si, Gyeongsangnam-do, Republic of Korea; c Department of Biotechnology, The Catholic University of Korea, Bucheon-si, Gyeonggi-do, Republic of Korea; d Microbiome Initiative, Yonsei University, Seoul, Republic of Korea; University of Greifswald

**Keywords:** systems biology, origin of replication, chromosomal landscape, transcriptome optimization, evolutionary adaptability

## Abstract

Adaptation through the fitness landscape may be influenced by the gene pool or expression network. However, genetic factors that determine the contribution of beneficial mutations during adaptive evolution are poorly understood. In this study, we experimentally evolved wild-type Escherichia coli K-12 MG1655 and its isogenic derivative that has two additional replication origins and shows higher background fitness. During the short time of experimental evolution, the fitness gains of the two E. coli strains with different fitness backgrounds converged. Populational genome sequencing revealed various mutations with different allele frequencies in evolved populations. Several mutations occurred in genes affecting transcriptional regulation (e.g., RNA polymerase subunit, RNase, ppGpp synthetase, and transcription termination/antitermination factor genes). When we introduced mutations into the ancestral E. coli strains, beneficial effects tended to be lower in the ancestor with higher initial fitness. Replication rate analysis showed that the various replication indices do not correlate with the growth rate. Transcriptome profiling showed that gene expression and gene ontology are markedly enriched in populations with lower background fitness after experimental evolution. Further, the degree of transcriptional change was proportional to the fitness gain. Thus, the evolutionary trajectories of bacteria with different fitness backgrounds can be complex and counterintuitive. Notably, transcriptional change is a major contributor to adaptability.

**IMPORTANCE** Predicting the adaptive potential of bacterial populations can be difficult due to their complexity and dynamic environmental conditions. Also, epistatic interaction between mutations affects the adaptive trajectory. Nevertheless, next-generation sequencing sheds light on understanding evolutionary dynamics through high-throughput genome and transcriptome information. Experimental evolution of two E. coli strains with different background fitness showed that the trajectories of fitness gain, which slowed down during the later stages of evolution, became convergent. This suggests that the adaptability of bacteria can be counterintuitive and that predicting the evolutionary path of bacteria can be difficult even in a constant environment. In addition, transcriptional change is associated with fitness gain during the evolutionary process. Thus, the adaptability of cells depends on their intrinsic genetic capacity for a given evolutionary period. This should be considered when genetically engineered bacteria are optimized through adaptive evolution.

## INTRODUCTION

Experimental evolution is a powerful tool to understand how an organism adapts to a specific environment (1). The adaptation of bacteria to a variety of environmental conditions has been examined to observe the dynamics within a population or the causality between genotype and phenotype (2–6). Experimental evolution has been conducted in various environmental conditions, including a minimal medium with a sole carbon source (5), high and low temperatures (6, 7), different light levels (8), and periodically changing temperatures (7). Furthermore, experimental evolution can be conducted during stationary phase or under the condition of protein overexpression (9, 10). Experimental evolution can be accomplished by the serial transfer of cells into a fresh medium, continuous culture with or without a chemostat, transfer of the colonies to another solid medium, or even without a fresh medium (8–10). During the evolutionary process, fitness increases as beneficial mutations accumulate (1). Predicting bacterial evolution can be difficult, but adaptive mutations during experimental evolution were recently collected and sorted in a database (11). The accumulation of knowledge on adaptive mutations allowed machine learning-based approaches to predict evolution (12, 13).

Most bacteria have a single circular chromosome with one origin of replication (*ori*). Similar to other bacteria, replication of the chromosome in Escherichia coli starts from *oriC* and extends bidirectionally. Even if an extra copy of the *ori* is inserted into the genome of E. coli, the original *ori* still functions well (14, 15). The position of *oriC* may be one of the major factors affecting the DNA replication and physiology of E. coli. Integration of an additional *oriC*-*mioC* in the *lacZ* gene and the *dadX*-*cvrA* intergenic region exerted a beneficial effect on growth as well as different replication properties in E. coli growing in M9 minimal medium (16).

In the present study, we utilized experimental evolution to investigate the effects of initial fitness (i.e., growth rate) on adaptability using a wild-type E. coli strain and its derivative with two additional *ori* (16). We measured the growth rate of evolved populations and sequenced their genomes. Furthermore, we introduced dominant mutations identified from populational genome sequencing to the ancestral strains. We calculated the replication rate at the population level to investigate the relationship between fitness gain and active DNA replication. Finally, we performed RNA sequencing (RNA-seq) to investigate the number of differentially expressed genes (DEGs), gene ontology (GO) enrichment, DEG clusters, and dissimilarities in global transcription patterns of evolved populations by comparing with ancestral strains.

## RESULTS

### Possible evolutionary trajectories of E. coli strains with different fitness backgrounds.

**FIG 1:**
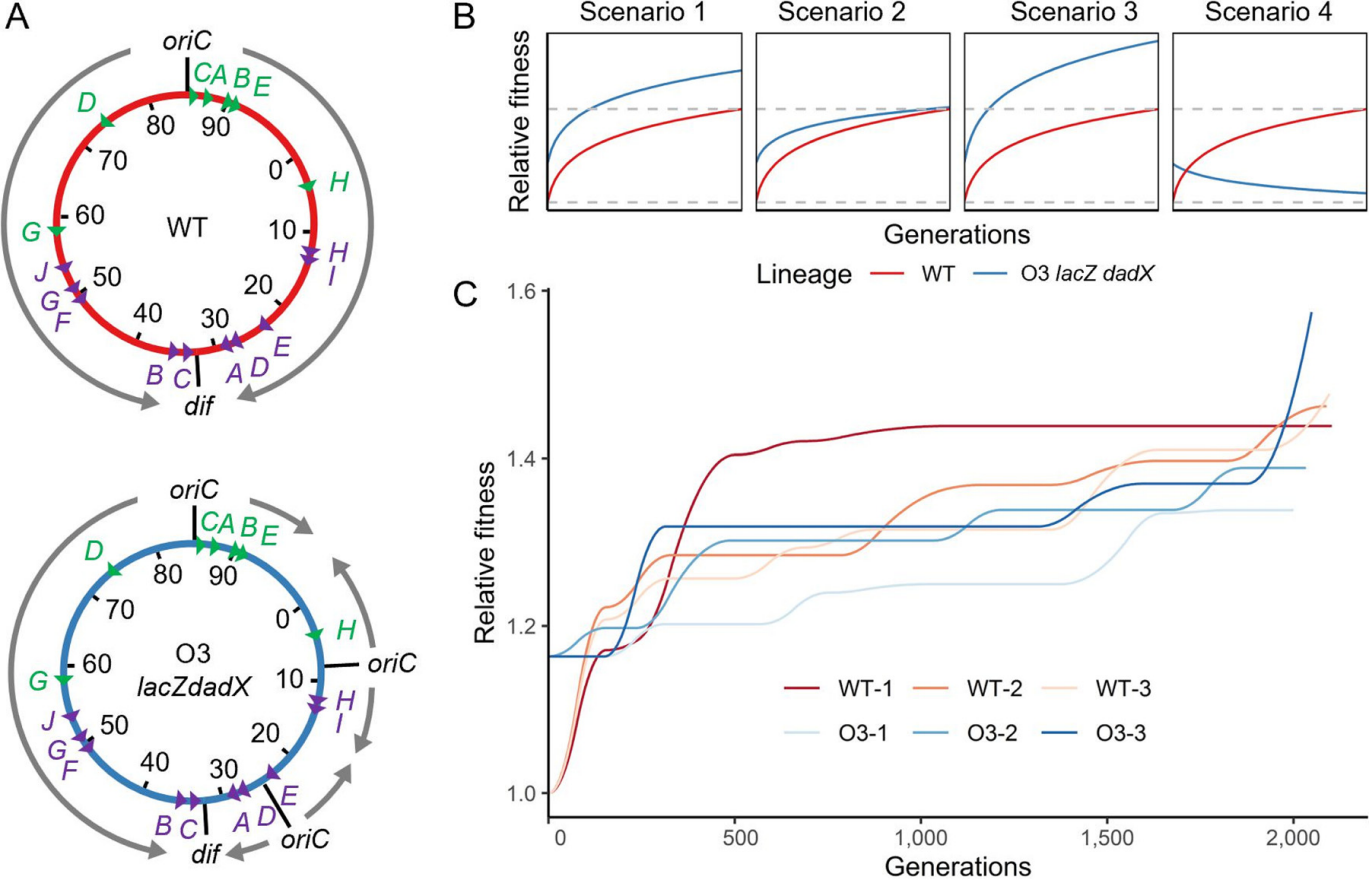
Experimental design and the trajectory of fitness changes during experimental evolution. (A) Illustration of the genomic architecture of two ancestral strains used for experimental evolution and the experimental scheme. Gray arrows, directions of replication; green triangles, rRNA operons; purple triangles, replication termination sites. (B) Possible fitness trajectory scenarios that can be assumed. Dashed gray lines indicate the initial and final fitness levels of WT E. coli during experimental evolution. (C) Changes in the relative fitness of the evolving populations. Relative fitness was calculated from the growth rate of the WT ancestor. Note that the relative fitness of ancestral O3 *lacZ dadX araA* is 1.16. WT, wild-type E. coli MG1655; O3, a derivative of E. coli MG1655 with two additional replication origins and an arabinose utilization marker.

We first defined fitness as growth rate and adaptability as fitness gain during a given time. Next, we established an E. coli K-12 MG1655 wild-type strain (WT) and its derivative with two additional *ori* (Fig. 1A) (E. coli K-12 MG1655 Δ*lacZ*::*oriC*-*mioC* Δ*dadX*/*cvrA*::*oriC*-*mioC*; referred to here as O3 *lacZ dadX*). This derivative grows faster than WT in a M9 minimal medium supplemented with 0.4% glucose as the sole carbon source (M9glc) (16). To demonstrate adaptability, we used an isogenic derivative of WT instead of different phylotypes or ecotypes of a species (e.g., MG1655, BL21, or W) or different taxa, to minimize issues stemming from the initial genetic background. If an ancestral strain harboring mutations that emerge during adaptive laboratory evolution is used, several subsequent mutations could be epistatic to those in the ancestral strain, and the fitness trajectory will follow suit (5). Moreover, the addition of *ori* is a novel mutation for fitness gain in bacteria that usually does not occur in nature.

During the evolution of WT and O3 *lacZ dadX*, several adaptation scenarios can be hypothesized (Fig. 1B). First, the adaptability may be similar between the two strains, which is the most plausible. Despite their different replication properties, the candidate mutations that can increase fitness may be similar. Furthermore, mutations in the two strains could have equivalent effects on fitness; thus, increases in fitness can be comparable. Second, the fitness gain of O3 *lacZ dadX* can be lower than that of WT. It can be assumed that introducing new *ori* into the chromosome of E. coli (which adapted to use only one replication origin with bidirectional replication long ago) may compromise replication physiology and, consequently, the bacteria may grow abnormally during evolution. Furthermore, due to the multiple replication systems, the pool of beneficial mutations can be reduced or completely changed; thus, the contribution of beneficial mutations to fitness can decrease, leading to a convergent fitness trajectory. Third, the adaptive potential can be higher in the O3 *lacZ dadX* strain. The relocation of *ori* may induce a conflict between the replication and transcription mechanisms in highly transcribed regions such as rRNA operons (17, 18). A head-on collision between the replication and transcription machinery can also increase the mutation rate and interrupt the progression of the replication fork, which is critical for balanced cell proliferation (19). Therefore, a new type of mutation that overcomes the replication stress can be induced. In other words, the pool of beneficial mutations can be expanded, and synergistic effects with mutations that appear in WT may occur. Fourth, the fitness of the O3 *lacZ dadX* population can be decreased after evolution. Since replication-transcription conflict increases the mutation rate, the insertion of multiple *ori* can escalate the mutation rate. A high mutation rate has been shown to limit adaptation (20). Some populations with the highest mutation rate showed abnormal fitness trajectories with consistent or decreased fitness after evolution. Therefore, fitness may decline, or constant fitness trajectories may be observed.

### Experimental evolution of E. coli strains with different DNA replication schemes.

Before experimental evolution, the wild-type *araA* gene in O3 *lacZ dadX* was replaced by a defective *araA* gene from E. coli B REL606 Ara^−^ to make it an Ara auxotroph. To avoid cross-contamination between the WT and O3 lineages, primers capable of checking the insertion of *oriC*-*mioC* in O3 *lacZ dadX araA* were constructed (Table S1). Upon insertion of the Ara^−^ marker, 16 genes (*yeaK* to *dmlA*) and partial sequences of *yeaJ* and *yeaV* were deleted, but this did not affect growth and competitive fitness (Fig. S1). Each strain evolved in parallel in three independent flasks containing M9glc for approximately 2,000 generations at 37°C (Fig. S2; WT-1, WT-2, WT-3, O3-1, O3-2, and O3-3 lineages), taking into account that genetic mutations that may substantially contribute to environmental fitness are substituted in the population during the early stages of evolution (2, 21). Cells were serially diluted once every 12 h to maintain their states in the exponential phase, since we used the growth rate as a criterion for fitness, and the growth rate of bacteria is best measured during the exponential phase, when the bacteria grow fastest. During the evolutionary process, the fitness of all populations was increased compared to that of the ancestral strains (Fig. 1C and Fig. S3). The populations that evolved in independent flasks followed different fitness trajectories during the evolutionary process. Although the experimental evolution was conducted for a similar number of generations, the fitness gain of the O3 populations at day 68 (68O3-1, 68O3-2, and 68O3-3) was generally lower than that of the WT populations at day 68 (68WT-1, 68WT-2, and 68WT-3) (one-sided *t*-test, *P < *0.001). Moreover, fitness gain during the first 33 days of evolution was significantly higher than that during the last 35 days of evolution (one-sided *t*-test, *P < *0.001).

### Chromosomal landscapes and genome dynamics during experimental evolution.

**FIG 2:**
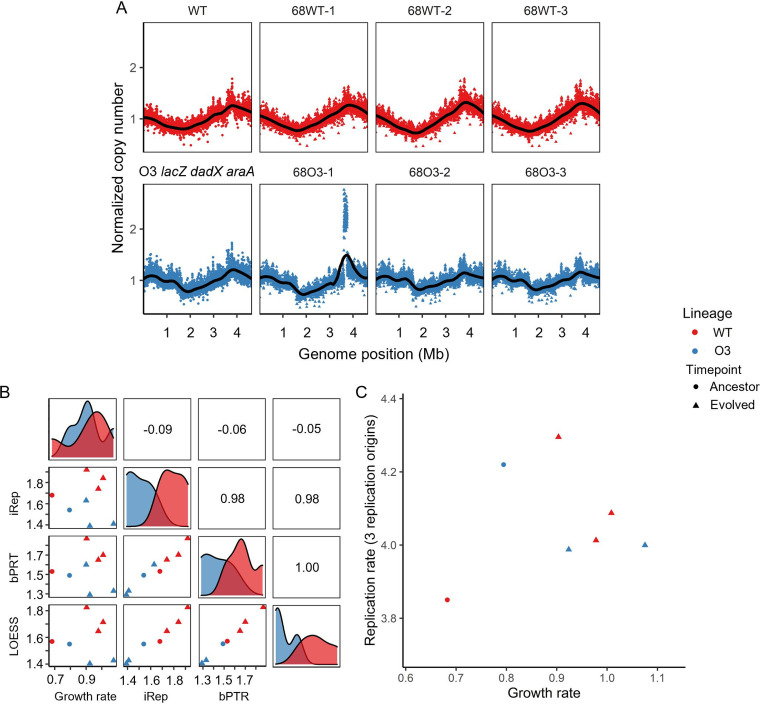
Replication profile of ancestral and evolved populations. (A) Replication profile of exponentially growing E. coli populations. The normalized copy number was calculated by dividing the average coverage of the 1,000-bp window by the total coverage of reads mapped to the genome. The black line indicates the LOESS smoothing line. (B) Pearson correlation plot between the replication rate calculated from the “original” replication origin. and the growth rate of E. coli populations. The 68O3-1 population was eliminated from the data. iRep, index of replication; bPTR, peak-to-trough ratio; LOESS, *ori*/*ter* ratio calculated from locally estimated scatterplot smoothing. (C) Correlation plot between growth rate and replication rate calculated from three replication origins. The 68O3-1 population was eliminated from the data. WT, wild-type E. coli MG1655; O3, a derivative of E. coli MG1655 with two additional replication origins and an arabinose utilization marker.

When additional *ori* sites are inserted into E. coli, replication happens simultaneously in several sites along the genome (14, 15). Thus, we explored the operation and activity of the additional *ori* inserted into the O3 strain through experimental evolution. We extracted genomic DNA (gDNA) from the cell populations in the exponential growth phase to obtain actively replicating genomes. Even after ~2,000 generations of experimental evolution, the replication pattern did not change in either WT or O3 (Fig. 2A). The chromosomal landscapes of the WT lineages exhibited a single peak and trough; the coverage patterns of the descendants of O3 *lacZ dadX araA* exhibited three peaks and three troughs each. A genome duplication seemed to have occurred between *rhsA* and *rhsB* in the 68O3-1 (sub)population based on the duplication of the normalized copy number.

Next, we measured the replication rate of the evolving populations, which increases as population growth increases (22). First, we calculated the replication rate using the “original” *ori* and the termination site. The normalized sequencing coverage was smoothed using locally estimated scatterplot smoothing (LOESS). Then, we compared the maximum-to-minimum ratio to the growth rate. To confirm the result, the index of replication (iRep) and peak-to-trough ratio (bPTR) were calculated using the iRep pipeline (22). Due to genome duplication between *rhsA* and *rhsB*, the 68O3-1 population was excluded when calculating the replication rate. Although three replication indices showed a significant correlation (correlation coefficient ≥ 0.98), the replication rate of the original *ori* was not significantly correlated with the growth rate (Fig. 2B). The iReps were also significantly lower in the O3 lineages (one-sided *t*-test, *P < *0.01). We predicted that the replication machinery such as DnaA or the DNA polymerases could be redistributed to three *ori* in the O3 lineages, which decreased the copy number of the original *ori*. Thus, we calculated the overall replication rate using three *ori*. The copy numbers at the three peak loci were summed and divided by the copy number at the termination site. In the case of WT lineages, the copy numbers at the *lacZ* and *dadX*/*cvrA* loci (at 365,000 and 1,240,000 bp on the chromosome, respectively) were used instead of the additional *ori*. However, the replication rate calculated from the copy numbers of three replication origins showed a weak correlation between the replication rate and the growth rate (Fig. 2C).

We then analyzed the genomes at the population level to investigate the evidence for beneficial mutations and casualties of the different evolutionary aspects between the WT and O3 lineages. To observe the overall population dynamics throughout the evolutionary processes, we compared the population genome sequences of the ancestors and their descendants that evolved for 2,000 to 2,102 generations. Variants in the genomes were identified using the breseq pipeline (23, 24). Only mutations with allele frequencies of over 10% were used to remove sequencing artifacts. Single-nucleotide polymorphisms (SNPs) and deletions, insertions, and other polymorphisms (DIPs) that had ≥50% allele frequencies are summarized in Table 1. Overall, 25 SNPs and 21 DIPs, including one large structural variation (duplication), occurred. Among the SNPs in the protein-coding genes, 20 and 3 mutations were missense and nonsense, respectively. Synonymous mutations at ≥10% allele frequencies were not found throughout the experimental evolution. Mutations in *pyrE-rph* and *nagA* were common among some sequenced populations. However, no specific mutation occurred in all populations. At the gene level, mutations commonly occurred in *ptsP*, *rpoB*, *nusA*, and the upstream region of *yobF* in several lineages. During the evolutionary process, approximately 35.6% of the mutations were fixed in the populations (Table S2).

**TABLE 1:** SNPs and DIPs identified in the evolving populations^*a*^

Lineage and gene	Annotation	Mutation	Allele frequency (%)	Description
WT-1				
*mrdB*	R69H (CGC→CAC)	C→T	100	Cell wall shape-determining protein
*pyrE-rph*		Δ82 bp	100	Orotate phosphoribosyltransferase-ribonuclease PH (defective); enzyme; degradation of RNA; RNase PH
*rpoB*	A1055V (GCG→GTG)	C→T	100	RNA polymerase, beta subunit
WT-2				
*cysE*	S224A (TCC→GCC)	A→C	100	Serine acetyltransferase
*rpoC*	R1075C (CGT→TGT)	C→T	100	RNA polymerase, beta prime subunit
WT-3				
*rlmH*	S121* (TCG→TAG)	G→T	100	23S rRNA m(3)Psi1915 pseudouridine methyltransferase, SAM dependent
*ydhZ*-*pykF*	Intergenic (−284/−273)	Δ1 bp	100	Uncharacterized protein/pyruvate kinase I
*ybcL*		IS*2* (+) +5 bp	87.90	Inactive polymorphonuclear leukocyte migration suppressor; DLP12 prophage; UPF0098 family secreted protein
O3-1				
*pykF*	R385L (CGC→CTC)	G→T	100	Pyruvate kinase I
*gtrB*	S267L (TCA→TTA)	C→T	100	CPS-53 (KpLE1) prophage; bactoprenol glucosyl transferase
*rpoB*	I524M (ATT→ATG)	T→G	100	RNA polymerase, beta subunit
*spoT*	Y14D (TAC→GAC)	T→G	91.00	Bifunctional (p)ppGpp synthetase II/guanosine 3′,5′-bis(pyrophosphate) 3′-pyrophosphohydrolase
O3-2				
*yeiH*	I137F (ATC→TTC)	A→T	100	UPF0324 family inner membrane protein
*ptsP*	Q340* (CAG→TAG)	G→A	100	PEP protein phosphotransferase enzyme I; GAF domain containing protein
*nusA*	Coding (1457/1488 nt)	+G	100	Transcription termination/antitermination L factor
*pyrE*-*rph*	Intergenic (−33/+33)	Δ1 bp	100	Orotate phosphoribosyltransferase/ribonuclease PH (defective); enzyme; degradation of RNA; RNase PH
*nagA*	Coding (422/1149 nt)	Δ1 bp	51.60	*N*-Acetylglucosamine-6-phosphate deacetylase
O3-3				
*ptsP*	L111* (TTA→TGA)	A→C	100	PEP protein phosphotransferase enzyme I; GAF domain-containing protein
*mreB*	A82S (GCC→TCC)	C→A	100	Cell wall structural complex MreBCD, actin-like component MreB
*pyrE-rph*		Δ82 bp	100	Orotate phosphoribosyltransferase/ribonuclease PH (defective); enzyme; degradation of RNA; RNase PH
*ydcK*	Coding (299/981 nt)	Δ1 bp	68.90	Uncharacterized protein
*ydbA*	Pseudogene (21–29/2513 nt)	IS*1* (+) +9 bp	66.70	Pseudogene, autotransporter homolog; interrupted by IS2 and IS30
*cyaA*	R160L (CGC→CTC)	G→T	64.40	Adenylate cyclase

a Only mutations with an allele frequency greater than 50% are shown. SNPs, single nucleotide polymorphisms; DIPs, deletions, insertions and other polymorphisms; SAM, S-adenosyl methionine; PEP, phosphoenolpyruvate; GAF, cGMP-specific phosphodiesterases, adenylyl cyclases and FhlA.

### Effect of background fitness on the fitness shift following the introduction of mutations.

**FIG 3:**
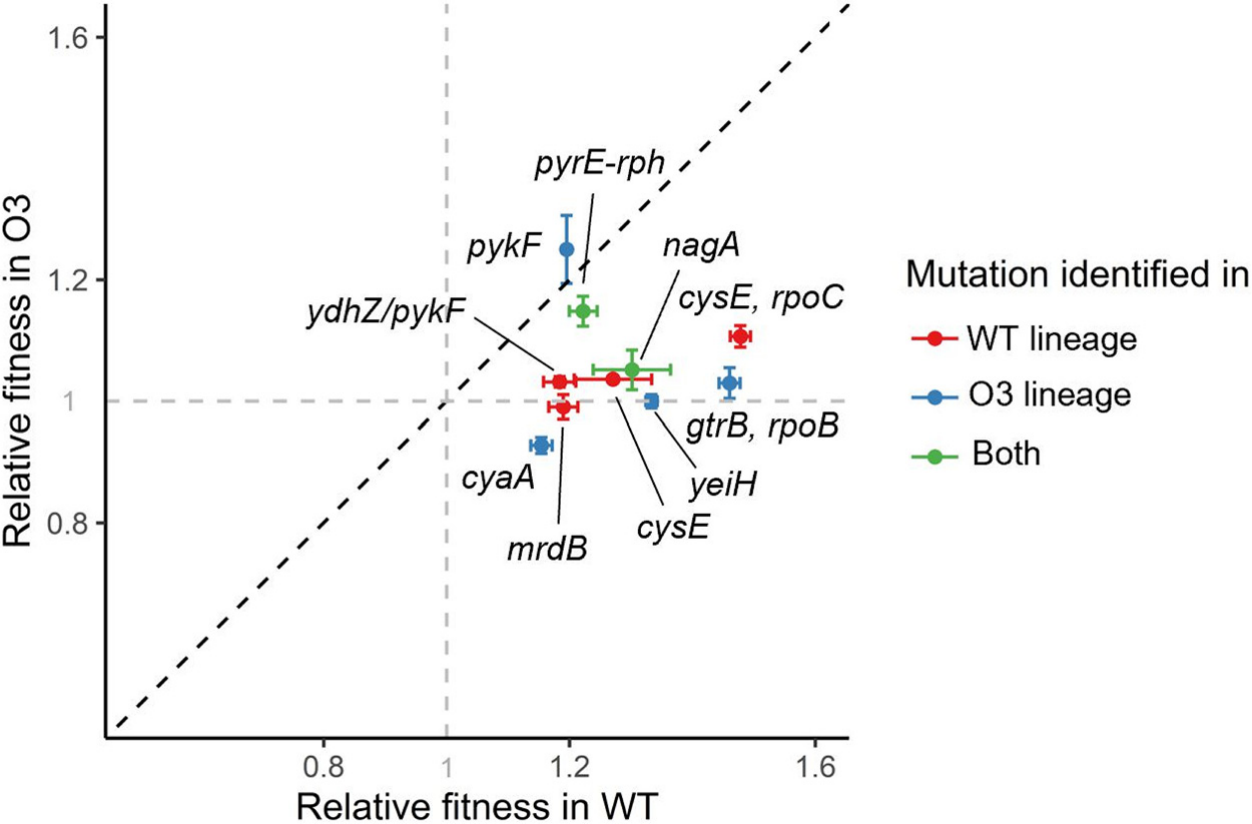
Fitness contribution of dominant mutations introduced to the ancestors individually or in combination. Relative fitnesses of WT and O3 *lacZ dadX* with introduced dominant mutations present in evolved populations are shown. Red, mutations identified in the WT lineage; blue, mutations identified in the O3 lineage; green, mutations identified in both lineages. Dashed gray line, relative fitness of 1. WT, wild-type E. coli MG1655; O3, a derivative of E. coli MG1655 with two additional replication origins. *mrdB*, mutation originating from 68WT-1; *cysE* and *cysE*/*rpoB*, mutations originating from 68WT-2; *ydhZ/pykF*, mutation originating from 68WT-3; *pykF* and *gtrB*/*rpoB*, mutations originating from 68O3-1; *yeiH*, mutation originating from 68O3-2; *cyaA*, mutation originating from 68O3-3; *pyrE*-*rph*, mutation originating from 68WT-1 and 68O3-3; *nagA*, mutation originating from 68WT-2 and 68O3-2.

Although O3 had high initial fitness, its adaptability was lower than that of WT. Thus, we used transient mutator multiplexed automated genome engineering (TM-MAGE) (25) to identify the fitness contribution of mutations for each ancestor. When we attempted to introduce the mutations into the O3 *lacZ dadX araA* strain, the efficiency of the method was very low—possibly due to the relationship between a defect in *araA* and the arabinose-inducible promoter in the recombination helper plasmid. Therefore, the mutant strain was prepared using the O3 *lacZ dadX* strain as a recipient. We built a total of 36 mutant strains. Mutations were introduced individually or in pairs or triplets into each of the two ancestral strains (Fig. S4). Furthermore, mutations identified in the WT lineages were inserted into O3 *lacZ dadX* and vice versa. We introduced 60.9% and 52.2% of the single mutations with over 50% allele frequency to WT and O3 *lacZ dadX*, respectively. When mutations were introduced into WT, all mutations increased the growth rate regardless of the lineage in which the mutation was identified (Fig. S4). In the O3 *lacZ dadX* background, 66.7% of the mutations showed beneficial effects on growth. When the same mutations were introduced into WT and O3 *lacZ dadX*, fitness gain tended to be lower in O3 *lacZ dadX* except in the case of the mutation in *pykF* (one-sided *t*-test, *P < *0.001) (Fig. 3). Furthermore, most of the confirmed beneficial mutations were not ancestor specific. These results show that fitness effects of the dominant mutation depend on the initial fitness of the ancestor (generally lower in O3). Thus, we conclude that the low adaptability of O3 *lacZ dadX araA* was caused by the low fitness contribution of each mutation compared to WT.

### The relationship between transcriptional optimization and adaptability.

**FIG 4:**
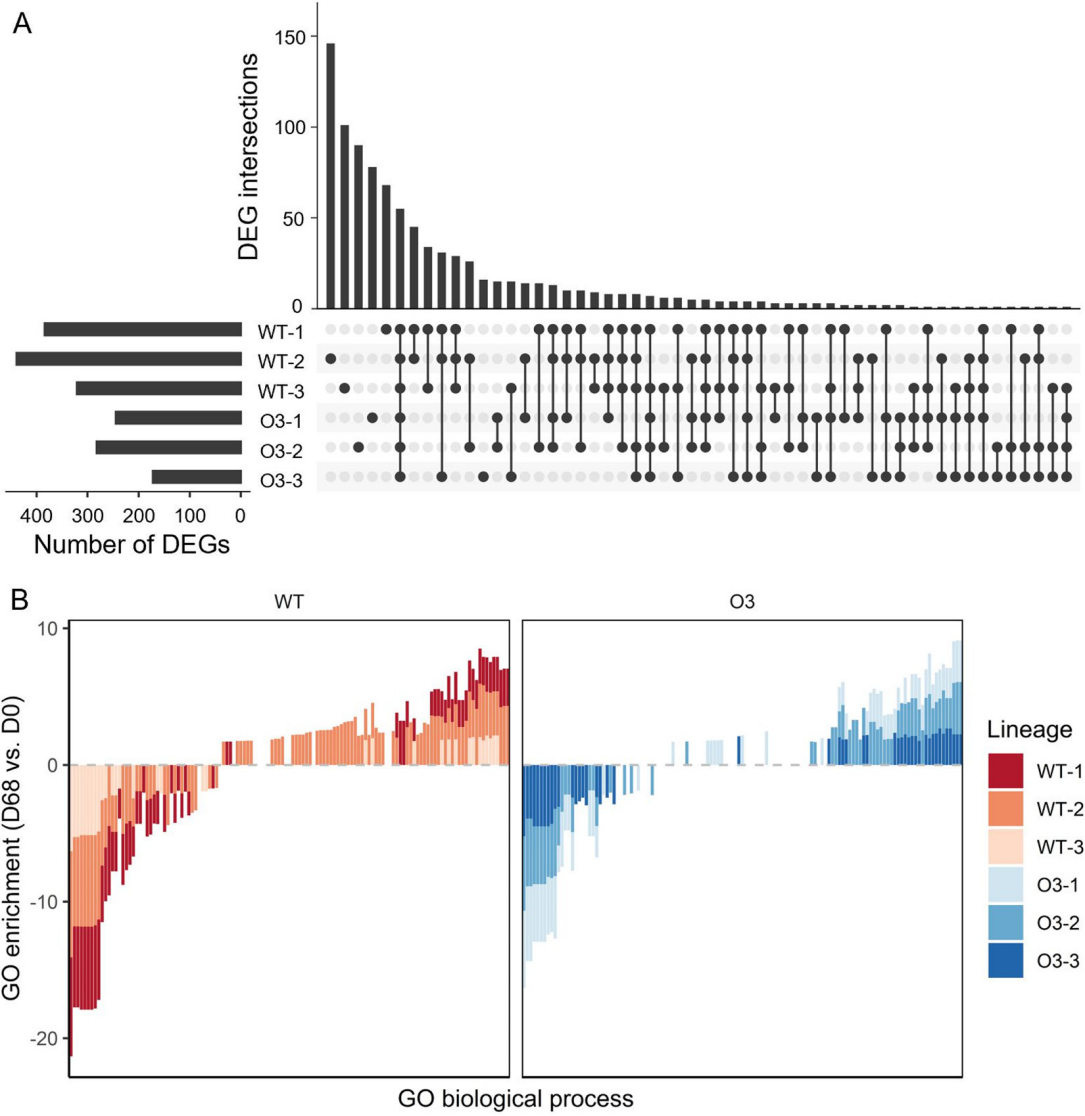
DEGs and GO analysis in the evolving populations. (A) UpSet plot of the numbers of significantly (*q *< 0.05 and |fold change| > 1.5) up- or downregulated genes of endpoint populations (day 68; 2,000 to 2,102 generations). (B) GO biological processes significantly (*P < *0.05) changed in WT or O3 endpoint populations. A GO enrichment value of >0 indicates upregulation, while a value of <0 indicates downregulation; the gray line shows a GO enrichment value of 0. WT, wild-type E. coli MG1655; O3, a derivative of E. coli MG1655 with two additional *ori* and an arabinose utilization marker.

A mutation in a regulatory gene during the evolutionary process affects the expression of other genes in addition to changes in functional activity (3). This phenomenon may have a greater effect when mutations occur in genes that influence global gene expression or translational effects. Mutation analysis showed that mutations in RNA polymerase subunits (*rpoB* and *rpoC*), rRNA methyltransferase (*rlmH*), and the transcription termination/antitermination protein (*nusA*) were fixed in the population (Table 1). Among the mutations that were not fixed in the population, many occurred in genes that play an important role in the transcriptional process, such as the upstream region of the DNA-binding transcription dual regulator (*hns*), GDP/GTP pyrophosphokinase (*relA*), and the bifunctional (p)ppGpp synthase/hydrolase (*spoT*) (Table S2). Thus, to investigate the global transcriptional change, we analyzed the populational transcriptome profile using RNA-Seq. For genes that were deleted during the insertion of the defective *araA* marker, the read count was regarded as zero. To compare the global transcription patterns and analyze the effect of the ancestral strain and evolutionary time point, we selected differentially expressed genes (DEGs) with a false discovery rate (*q* value) of <0.05 and |fold change| of >1.5, which is widely used in RNA-seq analysis (26–28). In WT endpoint populations, 328 and 392 genes were up- and downregulated, respectively, and 9.8% and 21.9% of the genes were commonly over- or underexpressed, respectively, among the three parallel populations (Fig. 4A). On the other hand, in O3 endpoint populations, the expression levels of 284 genes were increased and 210 decreased. Also, each population had 6.3% overexpressed and 22.4% underexpressed genes in common. Twenty-nine genes changed expression only in the WT populations, while two did so only in O3 (Table S3). In general, the number of DEGs with a significant difference was lower in the O3 lineage (one-sided *t*-test, *P < *0.05).

The mutational analysis showed that most mutations were not common between the WT and O3 lineages. Thus, we tried to find the underlying link between transcriptional change and phenotypic aspects. We analyzed the expression profile by grouping the gene function. We then used gene ontology (GO) to elucidate (i) underlying causes of the differential effects of the mutations and (ii) differential adaptabilities of the two strains with different genetic backgrounds. When we selected the commonly enriched GO terms between WT and O3 endpoint populations, 94.4% of GO enrichment tended to be higher in WT (Fig. 4B). Next, we analyzed the position-related expression patterns to identify the DEG clusters. A total of eight and seven DEG clusters were found in WT and O3 endpoint populations, respectively, compared to the ancestral strain as controls (Fig. S5). In the case of the WT lineages that evolved for 68 days, three DEG clusters were upregulated and five downregulated. For the O3 endpoint populations, three clusters were overexpressed and four were underexpressed.

**FIG 5:**
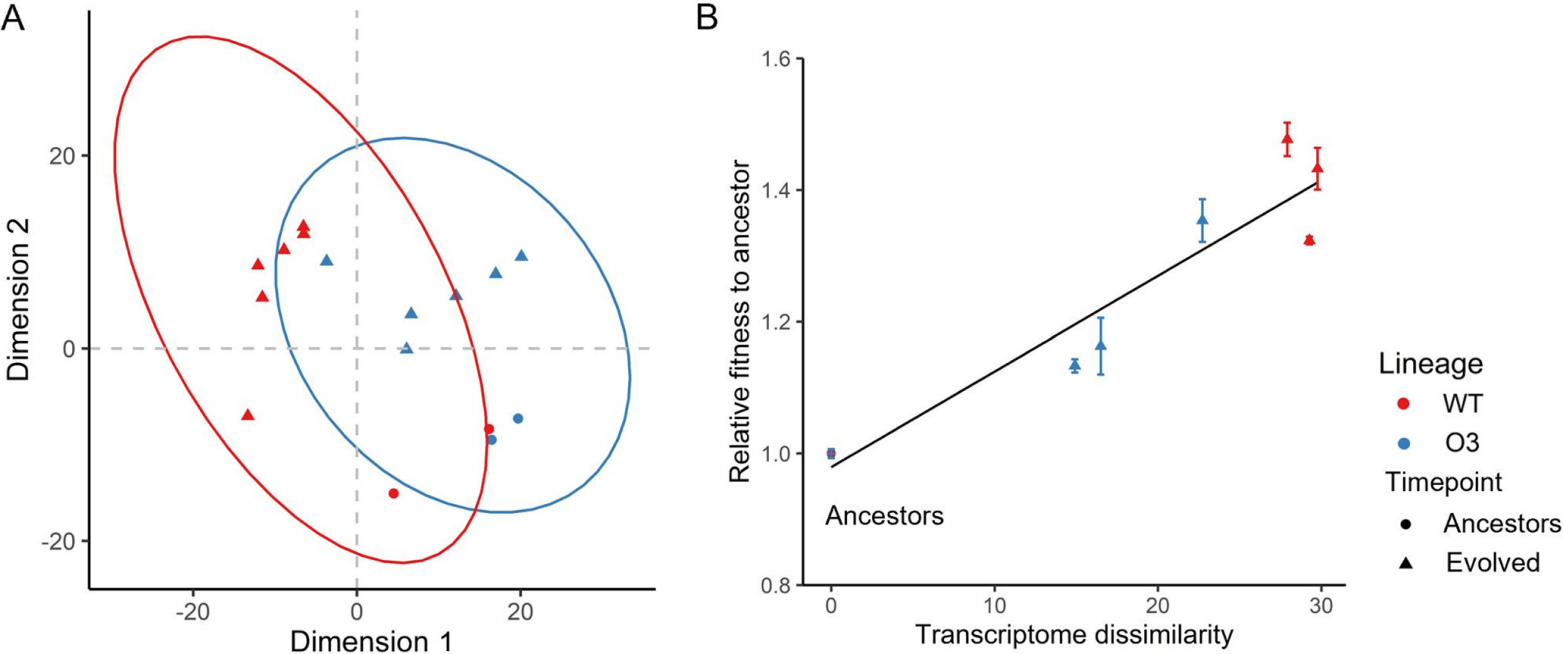
Relationship between transcriptome dissimilarity and relative fitness. (A) MDS (two-dimension) plot of global transcription patterns of ancestral and evolved populations. The MDS plot was generated using regularized log transformation of expression data in DESeq2. (B) Correlation plot between transcriptome dissimilarity and relative fitness. Transcriptome dissimilarity between populations was calculated by measuring the Euclidian distance between centroids on three-dimension MDS. Black line, linear regression line of two variables. WT, wild-type E. coli MG1655; O3, a derivative of E. coli MG1655 with two additional replication origins and an arabinose utilization marker.

Through these results, we assumed that the degree of transcriptional change may explain the adaptability of the evolving populations. Thus, we observed the global transcription pattern and calculated the transcriptome dissimilarity using multidimensional scaling (MDS), because the distance between the points in the MDS space is approximately the same as the dissimilarity (29). MDS showed the different global transcription patterns between WT and O3 (Fig. 5A). In addition, we demonstrated the relationship between fitness gain and transcriptome change. The distance between the two centroid points (two replicates) was used to calculate the dissimilarity between populations. The result calculated from MDS showed that the dissimilarity between the transcriptomes of each population and the ancestral strain was significantly proportional to the relative fitness (Fig. 5B) (linear regression; *R*^2^ = 0.8964, *P < *0.001).

## DISCUSSION

We experimentally evolved two E. coli strains, WT and O3 *lacZ dadX*, with different fitness backgrounds for ~2,000 generations and performed genomic and transcriptomic analyses on the evolved populations. Their adaptability was lower in the O3 lineages, which had a higher initial background fitness than WT. While population genome sequencing showed that they shared only a few dominant mutations, the TM-MAGE experiment demonstrated that the difference in adaptability stems from differential effects of the same mutation on the fitness of the ancestors. Transcriptome analysis further revealed that the rate of transcription change correlates to this phenotypic aspect.

Evolutionary biology often uses several criteria to measure fitness. Measuring relative proportions of the number of bacteria after cocultivation is the most common method to calculate competitive fitness during experimental evolution. When cells are exposed to toxic materials (e.g., antibiotics), the MIC can be measured to calculate fitness. Then, a strain with higher resistance to an antibiotic is considered to have a fitter genotype (30, 31). We measured the environmental fitness of the evolving E. coli populations using the growth rate because it is an accessible method for measuring fitness using a large number of populations. However, there are limitations when growth rate is used as a measure of fitness, because it is only a proxy of environmental fitness. Since the number of cells is not counted, the growth rate can be used only as an indirect indicator of competitive fitness. However, under our experimental conditions, cells were maintained in the exponential phase during the evolutionary process. Thus, the growth rate, which was measured in the exponential phase, can moderately reflect environmental fitness.

According to a previous study that tracked the fitness trajectory during 20,000 generations of long-term experimental evolution, relative fitness increased sharply before ~2,000 generations and then deaccelerated (2). As a result, we assumed that major mutations that contribute significantly to fitness appear before approximately 2,000 generations. However, we found only a limited number of common mutations during experimental evolution. Also, we did not observe any ancestor-specific mutations. If the number of replicates is increased or the experimental evolution is conducted for a longer time, it can be assumed that the common mutation can occur in a specific lineage.

Previous studies attempted to predict the growth of bacteria from next-generation sequencing data. For example, the rate of chromosome replication can be regarded as a readout for the growth rate of E. coli (32). The ratio between DNA copy numbers near *oriC* and *ter*, which is termed PTR, correlates with the growth rate of the bacterial population (22, 33). They showed that the growth dynamics of the microbial community can be accurately predicted by the replication rate under multiple growth conditions. However, we discovered that this criterion does not accurately predict fitness gain during experimental evolution. In other words, brisk DNA replication is not a crucial factor for fitness gain during the evolutionary process.

On the other hand, transcriptome sequencing and *k*-nearest neighbor models were used to estimate the growth rate of various E. coli clones that evolved for 216 to 611 generations (13). The study used ancestral strains that show growth defects because of gene deletions related to various metabolic pathways. After experimental evolution, mutations in RNA polymerase and transcription elongation factor were considered key mutations for growth restoration. Thus, by analyzing the transcriptome changes during growth restoration, the growth of evolved clones could be predicted by RNA-Seq. However, that study analyzed global transcription patterns of *sup* strains, which evolved to restore their growth. Therefore, adaptability may not be predicted when experimental evolution is conducted using wild-type strains or strains with other mutations as ancestors.

A previous study introduced mutations on *pykF*, *spoT*, *rbs*, and *topA* into natural E. coli isolates and compared genetic distances, phenotypic distances, and growth rates with normalized relative fitness (34). This study used mutations that provided a growth benefit in a laboratory-evolved population. The beneficial effects of mutations tended to be lower in strains with higher background fitness. However, the difference in phylotype or ecotype can lead to changes in accessory genes (35). We assumed that if genome composition is significantly different, the fitness effect caused by a specific mutation could be distinct. We minimized the genetic background and focused only on the background fitness, using the O3 *lacZ dadX araA* strain as an ancestor. As a result, we found that the strain with higher background fitness showed lower fitness gain from the mutations identified through populational genome sequencing—even though the genetic variation between the two ancestors is significantly low.

We conducted experimental evolution using a strain with a higher growth rate by inserting additional *ori* into the WT. However, studies on the effects of mutations are not limited to beneficial mutations or the evolution of faster-growing strains. A synthetic cross-phylum gene replacement experiment showed that most mutant strains with lower growth rates may have higher adaptability during evolution (36). Furthermore, initial fitness affects the robustness caused by deleterious mutations (37). Generally, it can be assumed that the effect of both beneficial and deleterious mutations depends on the background fitness of the host.

During the construction of O3 *lacZ dadX araA*, genes spanning from *yeaJ* to *yeaV* were inadvertently deleted from the chromosome. However, the growth rate and competitive fitness of O3 *lacZ dadX* and O3 *lacZ dadX araA* were identical. These genes are related to cyclic di-GMP synthesis (*yeaJ* and *yeaP*), conditions increasing mischarged aminoacyl-tRNA (*yeaK*), 2-nitroimidazole resistance (*yeaM* and *yeaN*), leucine analog resistance (*yeaS*), d-malate utilization (*yeaT* and *yeaU*), and a putative transporter (*yeaV*). However, the functions of *yoaL*, *yeaL*, *yeaO*, *yoaF*, *yoaK*, *yoaJ*, *yeaQ*, and *yeaR* remain unknown. Thus, the functions of these genes may not be important in exponential growth in M9glc. There will be phenotypic differences between the two strains under specific environmental conditions (e.g., when exposed to 2-nitroimidazole or growing in an M9 minimal medium supplemented with d-malate as the sole carbon source). If experimental evolution is conducted using a strain without such deletions, the difference in the genetic background can be further minimized.

In summary, our study demonstrates that the adaptability of E. coli may correlate with the potential for transcriptional change and that the fitness contribution of each mutation can be affected by transcriptional dissimilarity. Furthermore, this potential is governed by the background fitness of the ancestor. Thus, our research uncovers another layer of mechanisms that influence adaptability during experimental evolution.

## MATERIALS AND METHODS

### Bacterial strains, plasmids, and oligonucleotides.

All strains, plasmids, and oligonucleotides used in this study are described in Tables S1 and S4. Wild-type E. coli K-12 MG1655 [F^−^ λ^−^ *ilvG rfb*-*50 rph*-*1 glpK*(G184T)] and O3 *lacZ dadX araA* strains were used as ancestor strains for experimental evolution. O3 *lacZ dadX* is a genetically manipulated E. coli K-12 MG1655 strain with an extra *oriC*-*mioC* region at the *lacZ* site and an intergenic region between *dadX* and *cvrA* (16). All oligonucleotides over 50 nucleotides (nt) were purified using polyacrylamide gel electrophoresis. Oligonucleotides for TM-MAGE were synthesized by Bioneer (Korea), and other oligonucleotides were synthesized by Macrogen (Korea).

### λRed-mediated arabinose marker integration.

The arabinose utilization marker was integrated using a λRed recombination system (38) with a modified protocol. For recombination, the strains were cultured in LB medium (10 g tryptone, 5 g yeast extract, and 10 g NaCl per L) (Becton, Dickinson, USA). A 2× YT medium (10 g tryptone, 10 g yeast extract, and 10 g NaCl per L) (Becton, Dickinson, USA) was used and 1.5% agar (Becton Dickinson, USA) was supplemented to the solid medium. A pKD46 plasmid was used to introduce the γ, β, and *exo* genes for homologous recombination. Chloramphenicol acetyltransferase (*cat*) and sucrose counterselection marker (*sacB*) genes were amplified in pKD3 and pDMS197 plasmid (39) (Ex Taq; TaKaRa Bio, Japan), respectively. Two cassettes were digested with BamHI (New England Biolabs, USA) and ligated using T4 ligase (Promega, USA). The counterselection cassette was cloned into a pTOP TA V2 plasmid (Enzynomics, South Korea). The *cat*-*sacB* cassette was amplified using primers with homologous arms containing 50 bp at both ends of the *araA* gene (Ex Taq; TaKaRa Bio, Japan). PCR products were gel purified, digested with DpnI (New England Biolabs, USA), repurified, and suspended in distilled water.

The O3 *lacZ dadX* strain carrying the pKD46 plasmid was grown overnight in 2 mL of LB medium supplemented with 100 μg/mL ampicillin (Sigma-Aldrich, USA) at 30°C. The cells were diluted (1:100) and cultured under the same conditions. When the optical density at 600 nm (OD_600_) reached 0.1, the recombination system was induced with 10 mM arabinose and grown to an OD_600_ of 0.4. Cells were made electrocompetent by washing four times with ice-cold distilled water and once with ice-cold 10% glycerol (Bioshop, Canada) and concentrated 100-fold. Electroporation was conducted using an electroporator (MicroPulser electroporator; Bio-Rad, USA) and a 0.2-cm gap cuvette (Gene Pulser Cuvette; Bio-Rad, USA) at 1.8 kV using 50 μL cells and 0.9 μg PCR product. Then, 2× YT medium (1 mL) was added to the cells and incubated for 3 h at 37°C. Half of the cells were spread onto the lysogeny agar (LA) medium supplemented with 20 μg/mL chloramphenicol (Sigma-Aldrich, USA) to select resistant transformants.

The integration of the *cat*-*sacB* cassette was verified by colony PCR (GoTaq; Promega, USA). The inactivated *araA* gene was amplified in the E. coli B REL606 strain, and recombination was conducted as described above. After transformation of the linear *araA* gene into O3 *lacZ dadX* Δ*araA*::(*cat*-*sacB*), deletion of the *cat*-*sacB* cassette in the genome was confirmed by spreading the cells onto the counterselection agar medium (10 g tryptone [Becton Dickinson, USA], 250 g sucrose [Sigma-Aldrich, USA], and 5 g yeast extract [Sigma-Aldrich, USA] per L) to select the transformants carrying the *araA* gene amplified from REL606. To confirm the genotype of O3 *lacZ dadX araA*, gDNA was extracted with a Wizard gDNA purification kit (Promega, USA), and the *araA* sequence was determined by Sanger sequencing (Macrogen, South Korea).

### Experimental evolution.

At the beginning of the evolutionary experiment, single colonies of WT and O3 *lacZ dadX araA* strain were grown to stationary phase in a 500-mL Erlenmeyer flask filled with 100 mL M9glc (the following components were sterilized separately and then added: 200 mL 5× M9 salt [33.9 g/L Na_2_HPO_4_, 15 g/L KH_2_PO_4_, 5 g/L NH_4_Cl, 2.5 g/L NaCl], 2 mL 1 M MgSO_4_, 0.1 mL 1 M CaCl_2_, 10 mL 40% d-glucose per L of medium). Each strain was inoculated in a 250-mL Erlenmeyer flask containing 25 mL M9glc, and the cultures were shaken at 200 rpm and 37°C. Cells were transferred to the next flask every 12 h. Experimental evolution was conducted for ~2,000 generations. During the experimental evolution, the size of the inoculum was reduced to half at the 2nd, 14th, 34th, and 41st transfers to maintain the exponential phase. Passed generations for each flask were calculated by measuring OD_600_ using the following formula: log_2_(final cell density/initial cell density).

The entire number of generations during the experimental evolution experiment was calculated by adding all passed generations in each culture cycle. Cells (700 μL) were mixed with 300 μL 50% glycerol once every 3 days and stored as frozen stock. To confirm the contamination of the populations, the primers specific to each strain were designed, and the contamination was confirmed every 3 days using the above-mentioned primer sets and the arabinose utilization marker.

### Tracking fitness trajectories.

The growth rate was measured every 6 transfers. The growth of each population was measured using a SPARK microplate reader (Tecan, Switzerland). Bacterial populations (10 μL) were inoculated into 3 mL M9glc, cultured for 15 h, washed once with phosphate-buffered saline (PBS), and diluted (1:100), and 200-μL cultures were aliquoted to a 96-well tissue culture plate (SPL Life Science, South Korea). The growth rate was measured in triplicate. Cells were incubated at 37°C under 1.5-mm orbital shaking conditions (360 rpm), and the OD_600_ was measured once every 30 min. GrowthRates (40) was used to determine the growth rates of bacterial populations. Relative fitness was calculated by dividing the fitness of the population by the fitness of the WT. The relative fitness curves were drawn by quadratic spline interpolation and constrained to be monotonically increasing using the cobs package in R (version 1.3-3) (41, 42).

### Populational DNA sequencing and variant detection.

Frozen stock (10 μL) was inoculated into 3 mL M9glc and cultured to the exponential phase in aerobic growth condition at 37°C. The cells were washed once with PBS, reinoculated into 25 mL M9glc (OD_600_ ~ 0.02), and cultured to the exponential phase (OD_600_ ~ 0.4). Cells were centrifuged at 5,000 × *g* for 10 min at 4°C, and populational DNA was prepared using a Wizard genomic DNA purification kit (Promega, USA) according to the manufacturer’s instruction. Briefly, cells were resuspended with nucleus lysis solution and lysed by incubation at 80°C for 5 min. The cell lysate was treated with RNase A and incubated at 37°C for 30 min. Protein was precipitated with protein precipitation solution, and the supernatant containing populational DNA was purified by isopropanol/ethanol precipitation. DNA was rehydrated with a rehydration solution.

Illumina sequencing (Illumina, San Diego, CA, USA) was conducted by an external service (Macrogen, South Korea). Sequencing samples were prepared according to the Illumina protocols. The DNA quantity and quality were measured with PicoGreen (Thermo Fisher Scientific, USA). Briefly, 200 ng gDNA for a 550-bp insert size was fragmented by Covaris (Covaris, USA). The fragmented DNA was blunt-ended, phosphorylated, and end repaired. The appropriate library size was selected using different ratios of the sample purification beads. A single A was ligated to the 3′ end, and Illumina adapters were then ligated to the fragments. The final ligated product was then quantified using quantitative PCR (qPCR) according to the qPCR quantification protocol guide (KAPA Biosystems, USA), and quality was checked using the 4200 TapeStation (Agilent Technologies, USA). The sequencing-by-synthesis cycle was repeated to achieve the paired-end read length of 2 × 100 bp, and sequencing was carried out with the HiSeq 2500 platform (Illumina, USA). The quality of DNA sequencing reads is described in Table S5 and Fig. S6. Variants were found through the breseq (version 0.26.0) pipeline using polymorphism mode (population samples) in the pipeline (23, 24).

### Replication profile analysis.

Raw sequencing reads were imported into CLC Genomics Workbench 11.0 (Qiagen, Germany) and trimmed by using a quality score of 0.05 and a maximum number of ambiguities of two. Automatic read-through adapter trimming was used. For iRep and bPTR, trimmed reads were exported to .fastq files and mapped to the ancestral genome sequence by bowtie2 (43) (version 2.2.3) with the reorder function. iRep (22) (version 1.10) was used to determine the iRep and bPTR of the populations. For coverage analysis, trimmed sequencing reads were mapped to their ancestral genome sequence. The .csv files from mapping data were exported and the average coverage was calculated every 1,000 bp. To find peaks and troughs in the chromosomal landscape, LOESS was performed in RStudio (version 3.5.3; span = 0.3). Thereafter, LOESS maximum and minimum values were used as the copy number of the “original” replication origin and termination site. In the case of additional replication origins, a LOESS maximum between 0 to 1 Mb and 1 to 2 Mb was used as the copy number of additional replication origins.

### Mutant construction.

Oligonucleotides for TM-MAGE and the multiplex allele-specific colony PCR (MASC-PCR) were designed with the MODEST oligonucleotide design tool, with the length set to 90 bp and other options kept as default (44). A single colony each of WT and O3 *lacZ dadX* was transformed with plasmid pMA7-*sacB*. O3 *lacZ dadX* pMA7-*sacB* was inoculated into 3 mL LB Lennox medium supplemented with 0.5 mM MgSO_4_ and 100 μg/mL ampicillin (LLMA) and cultured overnight at 37°C with shaking at 200 rpm. The cells were diluted (1:100) in 15 mL medium and cultured to the exponential phase (OD_600_ ~ 0.4) under the same conditions. Then, *dam* methylase and recombinase were induced by the addition of l-arabinose to a final concentration of 0.2% (wt/vol) and incubated for 10 min. Cells were transferred to ice, incubated for 15 min, and washed three times with 30 mL, 10 mL, and 10 mL distilled water. Cells were resuspended with 1 mL distilled water, transferred to 1.5-mL microcentrifuge tubes, and centrifuged at 13,000 × *g* for 2 min. The supernatant was removed by pipetting; cells were resuspended in 200 μL distilled water, and 50 μL was transferred to a 0.2-cm gap cuvette (Gene Pulser Cuvette; Bio-Rad, USA). For each cycle, 0.5 μL of a 100-pmol/μL solution of oligonucleotides for mutagenesis was pooled by lineage and mixed. Electroporation was conducted by using an electroporator (MicroPulser Electroporator; Bio-Rad, USA) at 1.8 kV using 50 μL cells. After electroporation, 950 μL LLMA was added, and whole cells were inoculated into 14 mL LLMA. Cells were incubated to the exponential phase and moved to the next cycle. After four cycles of TM-MAGE, cells were grown overnight at 37°C and stored as frozen stock. The frozen stock was streaked onto counterselection agar medium, and MASC-PCR was conducted. For MASC-PCR, each primer concentration was mixed to 0.08 pmol/μL and used in the PCR (GoTaq; Promega, USA). The growth rate of each strain was measured in triplicate for each mutant isolate.

### Populational RNA sequencing.

Bacterial cells in the exponential phase were prepared under the same conditions as the populational DNA preparation. Before RNA extraction, cells were centrifuged at 13,000 × *g* for 1 min at 25°C, treated with RNAlater solution (Ambion, USA), and stored at −20°C. RNA was extracted using the RNeasy Mini Plus kit (Qiagen, Germany) according to the manufacturer’s instructions after chicken egg lysozyme (Sigma-Aldrich, USA) treatment for 5 min at 25°C. After RNA extraction, gDNA was eliminated with a Turbo DNA-free kit (Thermo Fisher Scientific, USA), and the remaining gDNA was confirmed by amplifying 16S rRNA genes using 27F and 1492R universal primers (GoTaq; Promega, USA).

Illumina sequencing was conducted by an external service (Macrogen, South Korea). The total RNA concentration was calculated using Quant-IT RiboGreen (Invitrogen, USA). To assess the integrity of the total RNA, samples were run on the TapeStation RNA ScreenTape (Agilent Technologies, USA). Only high-quality RNA preparations with an RNA integrity number (RIN) of >7.0 were used for RNA library construction. A library was independently prepared with 1 μg total RNA for each sample using the Illumina TruSeq stranded mRNA sample prep kit (Illumina, USA) according to the manufacturer’s instructions. First, rRNA was removed from the total RNA using a NEBNext rRNA depletion kit (New England Biolabs, USA). Thereafter, the remaining mRNA was fragmented into small pieces using divalent cations under elevated temperature. The cleaved RNA fragments were copied into the first-strand cDNA using SuperScript II reverse transcriptase (Invitrogen, USA) and random primers. This was followed by second-strand cDNA synthesis using DNA polymerase I, RNase H, and dUTP. These cDNA fragments then went through an end repair process, the addition of a single A base, and then ligation of the adapters. The products were then purified and enriched with PCR to create the final cDNA library. The libraries were quantified using KAPA library quantification kits for Illumina sequencing platforms according to the qPCR quantification protocol guide (KAPA Biosystems, USA) and qualified using the TapeStation D1000 ScreenTape (Agilent Technologies, USA). Indexed libraries were then subjected to paired-end (2 × 100 bp) sequencing on an Illumina HiSeq 2500 system (Illumina, USA). The quality of RNA-seq reads is described in Table S5 and Fig. S7.

### Transcriptome data analysis.

Raw .fastq files were trimmed by a minimum read length of 100 bp using Trimmomatic (version 0.39), and a Phred quality score of over 33 was used (45). Paired-end read quality was confirmed by FastQC (version 0.11.3). Trimmed reads were mapped onto genome sequences of their ancestral strain using Bowtie2 (version 2.2.3) (43). HTSeq (version 0.6.1) was used to calculate the read count mapped to the gene (46). Normalization and analysis of DEGs were conducted using the DESeq2 package in R (version 1.22.2) (47). GO enrichment analysis was conducted using the GAGE package in R (version 2.32.1) (48). Results were filtered using *q* value of <0.05 and |fold change| of >1.5 for DEG and a *P* value of <0.05 for GO enrichment analysis. The result from DESeq2 was transformed by a regularized log (rlog) transformation and used in MDS. The classical MDS was generated by the cmdscale function in RStudio. Position-related gene expression was analyzed by WOPPER using a *q* value of <0.1 and without a separated-strand analysis (49).

### Data availability.

The sequences used in this study were deposited in the Sequence Read Archive under the BioProject number PRJNA753969, which comprises 14 FASTQ files for Illumina HiSeq paired-end reads of the genome sequences of the two ancestors and six evolved E. coli populations and 28 FASTQ files for Illumina HiSeq paired-end reads of the transcriptome sequences of the ancestors and the evolved E. coli populations.
