# Supplementary material for: Transcriptional Potential Determines the Adaptability of Escherichia coli Strains with Different Fitness Backgrounds
Source: Microbiol Spectr. 2022 Nov 29;10(6):e02528-22. doi: 10.1128/spectrum.02528-22 (PMC9769844; doi:10.1128/spectrum.02528-22)
Supplement: Supplemental file 1 — Supplemental material. Download spectrum.02528-22-s0001.pdf, PDF file, 1.0 MB [file spectrum.02528-22-s0001.pdf]

Transcriptional potential determines the adaptability of *Escherichia coli* strains with different fitness backgrounds

Running title: Transcriptional potential determines the adaptability of *Escherichia coli*

Kitae Kim[a], Soon-Kyeong Kwon[a,b], Pil Kim[c], Jihyun F. Kim[a,d]

[a]Department of Systems Biology, Division of Life Sciences, and Institute for Life Science and Biotechnology, Yonsei University, 50 Yonsei-ro, Seodaemun-gu, Seoul 03722, Republic of Korea

[b]Division of Applied Life Science (BK21), Gyeongsang National University, 501 Jinju-daero, Jinju-si, Gyeongsangnam-do 52828, Republic of Korea

[c]Department of Biotechnology, The Catholic University of Korea, 43 Jibong-ro, Bucheon-si, Gyeonggi-do 14662, Republic of Korea

[d]Microbiome Initiative, Yonsei University, 50 Yonsei-ro, Seodaemun-gu, Seoul 03722, Republic of Korea

Address correspondence to Jihyun F. Kim, jfk1@yonsei.ac.kr, or Soon-Kyeong Kwon, skkwon@gnu.ac.kr.

SUPPLEMENTAL MATERIAL

Supplemental material contains five supplementary tables and seven supplementary figures.

Table S1. Strains and plasmids used in this study.

Table S2. Extended view of the single nucleotide polymorphisms (SNPs), deletions, insertions, and other polymorphisms (DIPs) identified in the evolving populations.

Table S3. DEGs commonly up- or down-regulated in WT or O3 populations.

Table S4. Oligonucleotides used in this study**.**

Table S5. Summary statistics of DNA and RNA sequencing.

Figure S1. Phenotypic difference between O3 *lacZ dadX* and O3 *lacZ dadX araA***.**

Figure S2. Experimental design of experimental evolution.

Figure S3. Growth curves of ancestral and evolved populations.

Figure S4. Effect of dominant mutations on fitness.

Figure S5. Position-dependent differentially expressed gene (DEG) cluster analysis.

Figure S6. DNA sequencing read quality after read trimming.

Figure S7. RNA sequencing read quality after read trimming.

SUPPLEMENTAL TABLES

Table S1. Strains and plasmids used in this study.

Strains, plasmids	Relevant characteristics	Source
**Strains**		
WT	*Escherichia coli* K-12 MG1655 wild type	Laboratory stock
O3 *lacZ dadX*	*E. coli* K-12 MG1655 Δ*lacZ*::*oriC-mioC* Δ*dadX/cvrA*::*oriC-mioC*	(1)
O3 *lacZ dadX araA*	O3 *lacZ dadX,* REL606 *araA,* Δ*yeaJ-yeaV*	This study
REL606	Ara$^-$	Laboratory stock
68WT-1	Evolved WT lineage 1 at day 68	This study
68WT-2	Evolved WT lineage 2 at day 68	This study
68WT-3	Evolved WT lineage 3 at day 68	This study
68O3-1	Evolved O3 lineage 1 at day 68	This study
68O3-2	Evolved O3 lineage 2 at day 68	This study
68O3-3	Evolved O3 lineage 3 at day 68	This study
WT *mrdB*	WT *mrdB* (R69H)	This study

WT *rpoB*	WT *rpoB* (A1055V)	This study
WT *mrdB rpoB*	WT *mrdB* (R69H), *rpoB* (A1055V)	This study
WT *rpoB pyrE-rph*	WT *rpoB* (A1055V), *pyrE-rph* (Δ82)	This study
WT *cysE*	WT *cysE* (S224A)	This study
WT *rpoC*	WT *rpoC* (R1075C)	This study
WT *cysE rpoC*	WT *cysE* (S224A), *rpoC* (R1075C)	This study
WT *rlmH*	WT *rlmH* (S121*)	This study
WT *ydhZ/pykF*	WT *ydhZ/pykF* (intergenic (-284/-273) Δ1)	This study
WT *pykF*	WT *pykF* (R385L)	This study
WT *gtrB rpoB*	WT *gtrB* (S267L), *rpoB* (I524M)	This study
WT *pyfK rpoB*	WT *pyfK* (R385L), *rpoB* (I524M)	This study
WT *yeiH*	WT *yeiH* (I137F)	This study
WT *yeiH ptsP*	WT *yeiH* (I137F), *ptsP* (Q340*)	This study
WT *ptsP nusA*	WT *ptsP* (Q340*), *nusA* (coding (1457/1488 nt)+G)	This study
WT *ptsP*	WT *ptsP* (L111*)	This study
WT *mreB*	WT *mreB* (A82S)	This study

WT *pyrE-rph*	WT *pyrE-rph* (Δ82)	This study
WT *nagA*	WT *nagA* (coding (422/1149 nt) Δ1)	This study
WT *cyaA*	WT *cyaA* (R160L)	This study
WT *cyaA mreB*	WT *cyaA* (R160L), *mreB* (A82S)	This study
O3 *mrdB*	O3 *lacZ dadX mrdB* (R69H)	This study
O3 *pyrE-rph*	O3 *lacZ dadX pyrE-rph* (Δ82)	This study
O3 *cysE*	O3 *lacZ dadX cysE* (S224A)	This study
O3 *cysE rpoC*	O3 *lacZ dadX cysE* (S224A), *rpoC* (R1075C)	This study
O3 *ydhZ/pykF*	O3 *lacZ dadX ydhZ/pykF* (intergenic (-284/-273)Δ1)	This study
O3 *rlmH ydhZ/pykF*	O3 *lacZ dadX rlmH* (S121*) *ydhZ/pykF* (intergenic (-284/-273)Δ1)	This study
O3 *gtrB*	O3 *lacZ dadX gtrB* (S267L)	This study
O3 *pykF*	O3 *lacZ dadX pykF* (R385L)	This study
O3 *rpoB*	O3 *lacZ dadX rpoB* (I524M)	This study
O3 *gtrB rpoB*	O3 *lacZ dadX gtrB* (S267L), *rpoB* (I524M)	This study
O3 *pykF gtrB rpoB*	O3 *lacZ dadX pykF* (R385L), *gtrB* (S267L), *rpoB* (I524M)	This study
O3 *ptsP*	O3 *lacZ dadX ptsP* (Q340*)	This study

O3 *nagA*	O3 *lacZ dadX nagA* (coding (422/1149 nt) Δ1)	This study
O3 *yeiH*	O3 *lacZ dadX yeiH* (I137F)	This study
O3 *cyaA*	O3 *lacZ dadX cyaA* (R160L)	This study
**Plasmids**		
pKD46	Used for λ-red recombination, *bla, γ, β, exo* (Red recombinase), temperature-conditional origin of replication	(2)
pKD3	Template plasmid for chloramphenicol acethyltransferase (CAT)	(2)
pDMS197	Template plasmid for *sacB* (sucrose counter selection marker)	(3)
pTOP TA V2	TOP cloning plasmid (Enzynomics)	
pTOP TA V2 CAT-*sacB*	Template plasmid for CAT-*sacB* selection-counter selection marker	This study
pMA7-SacB	Helper plasmid in transient mutator multiplex automated genome engineering (TM-MAGE) cycling	(4)

Table S2. Extended view of the single nucleotide polymorphisms (SNPs), deletions, insertions, and other polymorphisms (DIPs) identified in the evolving populations.

Lineage / position	Gene	Mutation	Allele frequency	Annotation	Description
**WT-1**					
26,680	*ispH* →	G→C	10.9%	S135T (AGT→ACT)	4-hydroxy-3-methylbut-2-enyl diphosphate reductase, 4Fe-4S protein
601,873	*cusA* → / → *pheP*	T→C	10.2%	intergenic (+16/-86)	copper/silver efflux system, membrane component/phenylalanine transporter
666,108	*mrdB* ←	C→T	100%	R69H (CGC→CAC)	cell wall shape-determining protein
1,152,740	*fabF* →	T→G	35.9%	Y268D (TAT→GAT)	3-oxoacyl-[acyl-carrier-protein] synthase II
1,907,636	*yobF* ← / ← *yebO*	IS*1* (+) +9 bp	13.9%	intergenic (-45/+617)	DUF2527 family heat-induced protein/putative inner membrane protein
2,731,457	*rrsG* ← / ← *clpB*	A→T	17.0%	intergenic (-300/+143)	16S ribosomal RNA of rrnG operon/protein disaggregation chaperone
2,915,962	*barA* →	IS*1* (+) +9 bp	12.8%	coding (906-914/2757 nt)	hybrid sensory histidine kinase, in two-component regulatory system with UvrY
2,966,966	*ptsP* ←	A→C	17.3%	F490C (TTC→TGC)	PEP-protein phosphotransferase enzyme I; GAF domain containing protein
3,815,859	*pyrE-rph*	Δ82 bp	100%		*pyrE-rph*

4,184,408	*rpoB* →	C→T	100%	A1055V (GCG→GTG)	RNA polymerase, beta subunit
**WT-2**					
696,419	*ubiF* → / ← *glnX*	+TTT	13.0%	intergenic (+143/+11)	2-octaprenyl-3-methyl-6-methoxy-1,4-benzoquinol oxygenase/tRNA-Gln
702,330	*nagA* ←	Δ1 bp	20.7%	coding (422/1149 nt)	N-acetylglucosamine-6-phosphate deacetylase
973,367	*elyC* ←	Δ1 bp	25.7%	coding (35/780 nt)	envelope biogenesis factor; DUF218 superfamily protein
1,019,695	*ompA* ←	C→T	29.2%	G120D (GGC→GAC)	outer membrane protein A (3a;II*;G;d)
1,293,196	*hns* ← / → *tdk*	IS*5* (+) +4 bp	40%	intergenic (-274/-328)	global DNA-binding transcriptional dual regulator H-NS/thymidine kinase/deoxyuridine kinase
2,311,785	*ompC* ←	+CAACAT	19.0%	coding (965/1104 nt)	outer membrane porin protein C
2,912,408	*relA* ←	A→C	14.7%	V415G (GTC→GGC)	(p)ppGpp synthetase I/GTP pyrophosphokinase
2,913,235	*relA* ←	T→G	11.0%	L139F (TTA→TTC)	(p)ppGpp synthetase I/GTP pyrophosphokinase
3,696,149	*yhjR* ←	C→T	27.9%	A13T (GCG→ACG)	DUF2629 family protein
3,781,893	*cysE* ←	A→C	100%	S224A (TCC→GCC)	serine acetyltransferase
4,188,572	*rpoC* →	C→T	100%	R1075C (CGT→TGT)	RNA polymerase, beta prime subunit
4,627,797	*nadR* →	IS*1* (+) +9 bp	14.2%	coding (483-491/1233 nt)	nicotinamide mononucleotide adenylyltransferase, ribosylnicotinamide kinase, transcriptional repressor
**WT-3**					

571,172	*ybcL* →	IS2 (+) +5 bp	87.9%	coding (280-284/552 nt)	inactive polymorphonuclear leukocyte migration suppressor; DLP12 prophage; UPF0098 family secreted protein
668,354	*rlmH* ←	G→T	100%	S121* (TCG→TAG)	23S rRNA m(3)Psi1915 pseudouridine methyltransferase, SAM-dependent
1,755,425	*ydhZ* ← / → *pykF*	Δ1 bp	100%	intergenic (-284/-273)	uncharacterized protein/pyruvate kinase I
1,915,319	*proQ* ←	+T	23.8%	coding (216/699 nt)	RNA chaperone, putative ProP translation regulator
**O3-1**					
1,756,482	*pykF* →	G→T	100%	R385L (CGC→CTC)	pyruvate kinase I
2,459,131	*gtrB* →	C→T	100%	S267L (TCA→TTA)	CPS-53 (KpLE1) prophage; bactoprenol glucosyl transferase
3,812,557	*spoT* →	T→G	91.0%	Y14D (TAC→GAC)	bifunctional (p)ppGpp synthetase II/ guanosine-3',5'-bis pyrophosphate 3'-pyrophosphohydrolase
4,172,934	*rpoB* →	T→G	100%	I524M (ATT→ATG)	RNA polymerase, beta subunit
**O3-2**					
701,363	*nagA* ←	Δ1 bp	51.6%	coding (422/1149 nt)	N-acetylglucosamine-6-phosphate deacetylase
2,240,243	*yeiH* →	A→T	100%	I137F (ATC→TTC)	UPF0324 family inner membrane protein
2,957,535	*ptsP* ←	G→A	100%	Q340* (CAG→TAG)	PEP-protein phosphotransferase enzyme I; GAF domain containing protein
3,306,188	*nusA* ←	+G	100%	coding (1457/1488 nt)	transcription termination/antitermination L factor

3,805,919	*pyrE* ← / ← *rph*	Δ1 bp	100%	intergenic (-33/+33)	orotate phosphoribosyltransferase/ribonuclease PH (defective);enzyme; Degradation of RNA; RNase PH
**O3-3**					
1,465,043	*ydbA* →	IS*1* (+) +9 bp	66.7%	pseudogene (21-29/2513 nt)	pseudogene, autotransporter homolog; interrupted by IS2 and IS30
1,499,782	*ydcK* ←	Δ1 bp	68.9%	coding (299/981 nt)	uncharacterized protein
1,897,746	*yobF* ← / ← *yebO*	IS*2* (+) +5 bp	24.9%	intergenic (-37/+629)	DUF2527 family heat-induced protein/putative inner membrane protein
2,958,221	*ptsP* ←	A→C	100%	L111* (TTA→TGA)	PEP-protein phosphotransferase enzyme I; GAF domain containing protein
3,307,050	*nusA* ←	G→A	32.3%	R199C (CGT→TGT)	transcription termination/antitermination L factor
3,390,962	*mreB* ←	C→A	100%	A82S (GCC→TCC)	cell wall structural complex MreBCD, actin-like component MreB
3,805,977	*pyrE*-*rph*	Δ82 bp	100%		*pyrE-rph*
3,981,749	*cyaA* →	G→T	64.4%	R160L (CGC→CTC)	adenylate cyclase
4,171,929	*rpoB* →	T→A	25.8%	D189E (GAT→GAA)	RNA polymerase, beta subunit
4,533,728	*fimA* →	Δ1 :: IS*186* (–) +6 bp :: Δ1	36.8%	coding (494-499/549 nt)	major type 1 subunit fimbrin (pilin)

Table S3. DEGs that are commonly up- or down-regulated in WT or O3 populations.

Lineage/gene	Function	Average Log_2FC	Average *q*-value
WT			
ydaM	diguanylate cyclase DgcM	-1.68896	0.000818
amn	AMP nucleosidase	-0.80977	0.008372
rnhB	RNase HII	0.709147	0.007159
yqfA	transmembrane homeostasis protein A	-1.50936	0.008587
ibaG	acid stress protein IbaG	0.635081	0.016929
srkA	stress response kinase A	0.741528	1.02E-05
mltA	membrane-bound lytic murein transglycosylase A	0.961929	5.35E-05
ybfA	DUF2517 domain-containing protein YbfA	-1.02759	0.013195
ybgA	DUF1722 domain-containing protein YbgA	-1.93694	0.018844
dnaG	DNA primase	0.815044	0.000232
csgG	curli secretion channel	-1.53791	0.002594
proP	osmolyte:H+ symporter ProP	-1.25239	0.015265

yqgC	protein YqgC	1.006299	0.022184
yaaU	putative transporter YaaU	-1.26135	0.026508
yhjD	putative transporter YhjD	-1.08171	0.022303
dtpD	dipeptide:H+ symporter DtpD	1.187037	0.016882
dnaE	DNA polymerase III subunit α	0.73969	0.007525
cspD	DNA replication inhibitor CspD	-0.94156	0.005748
pbpC	peptidoglycan glycosyltransferase PbpC	0.728647	0.020088
gadC	L-glutamate:4-aminobutyrate antiporter	-1.45671	0.019172
rlmN	23S rRNA m2A2503 methyltransferase/tRNA m2A37 methyltransferase	0.752209	0.004434
ppiA	peptidyl-prolyl cis-trans isomerase A	0.715015	0.023499
glcB	malate synthase G	-0.76428	0.020917
wcaC	colanic acid biosynthesis galactosyltransferase WcaC	-1.55763	0.010894
dkgA	methylglyoxal reductase DkgA	-1.57133	0.015858
dps	stationary phase nucleoid component that sequesters iron and protects DNA from damage	-2.4165	0.01543
gadB	gadB	-1.96865	0.001756
accA	acetyl-CoA carboxyltransferase subunit α	0.688628	0.011405

wzc	protein-tyrosine kinase Wzc	-1.39008	0.023037
O3			
racR	Rac prophage; DNA-binding transcriptional repressor RacR	-0.79431	0.001077
yffB	putative reductase YffB	0.807003	0.003224

Table S4. Oligonucleotides used in this study.

Oligonucleotides	Sequence (5’ to 3’)
**Marker integration**	
cat_F	TGTAGGCTGGAGCTGCTTC
cat_BamH1_R	CGGGATCCCATATGAATATCCTCCTTAGTTCC
sacB_F	CTG ACA TGG GAA TTC TGA TCC
sacB_BamH1_R	GAA TAC GGT TAG CCA TTT GCC GGA TCC CG
araA_cat-sacB_F	ATGACGATTTTTGATAATTATGAAGTGTGGTTTGTCATTGGCAGCCAGCAGAGAAGCCCTTAGAGCCTC
araA_cat-sacB_R	TTAGCGACGAAACCCGTAATACACTTCGTTCCAGCGCAGCGCGTCTTTAACATATGAATATCCTCCTTAGTTCC
REL606_araA_F	ATGACGATTTTTGATAATTATGAAG
REL606_araA_R	TTAGCGACGAAACCCGTAAT
araA_conf_F	GCGCTTTGCTTATCCGGCCTAC
araA_conf_R	GAGCGCCGAACAACACTATCTTCC
cat_outward	CGGTCTGGTTATAGGTACATTGAGC
sacB_outward	CGCAAACGCTTGAGTTGCGCC

**Contamination check**	
MG1655_F	TTCGCAACACGATGATGAATCG
W3110_F	GCATAGCTCCACCATCTCTG
yjaA_R	AGGCAGCGTCTCCTGACAC
O3_lacZ_conf_F	GGTTGATGTCATGTAGCCAAATCGGG
O3_lacZ_conf_R	GAAATCCCGAATCTCTATCGTGCGG
27F	AGAGTTTGATCMTGGCTCAG
1492R	TACGGYTACCTTGTTACGACTT
**TM-MAGE**	
WT1_mrdB	GCGCAAATTCCTCCACaCGTTTATGAAGGCTGGGCCCCCTATCTCTATATCATCTGTATTATTTTGCTGGTGGCGGTAGATGCTTTCGGT
WT1O31O33_rph	CTCATCAGTCGCCTTAAAAATCAGTTTGCCAGCGCCGCCTTCTGCCGTCCCCTGCACTTCAATGATGCGCCCGTCTTCGGTCATCACTAC
WT1_rpoB	TGATCTTAGAAATTACACCCTTGTTACCGTGACGACCTGCCATCTTGTCACCAGGCTGGATACGGCGTTTAACCaCCAGATATACCTTAA
WT2_cysE	GTTGCAGCACCACGGcACCTGCGCCAATCTTCGCGCCGCGCCCAACTTCAATATTGCCGAGGATTTTCGCGCCCGCGCCAATCATCACAC
WT2_rpoC	GCATATCGGTACCTGGGATCAGAACGTCGTTACCCTGAGCATCAACGATTTTCAGTGCCGGACaCAGATCTTTACCACCTGCGGTACGTT
WT3_rlmH	CGACGTCAGTCTACTGATTGGCGGGCCTGAAGGGTTGTCGCCTGCCTGTAAAGCGGCGGCTGAGCAGAGCTGGTaGCTGTCGGCGCTTAC
WT3_rlmH2	AGGGTTGTCGCCTGCCTGTAAAGCGGCGGCTGAGCAGAGCTGGTaGCTGTCGGCGCTTACCCTCCCCCATCCGCTGGTTCGCGTGCTGGT

WT3_ydhZpykF	ACCTTTTCCCTGGAACGTTAAATCTTTGATAACAATTTATTGTCTAACAAGTTGTATATTTTTTGAAACGCTGTTTTGTTTTCCTTTTGG
O31_pykF	GGCGGTAAATCTGCTCtCGCAGTACGTAAATACTTCCCGGATGCCACCATCCTGGCACTGACCACCAACGAAAAAACGGCTCATCAGTTG
O31_gtrB	ATGGGGCGTGGATGATTTTAGATACTATCATATTTGGAAATGCTGTTAGGGGATATCCTTCACTACTTGTTTtAATACTGTTTTTAGGTG
O31_spoT	TCGTTAATCACAAAGCGGGTCGCCCTTGTATCTGTTTGAAAGCCTGAATCAACTGATTCAAACCgACCTGCCGGAAGACCAAATCAAGCG
O31_wecE	ACAAAGTCGCAATTACCGTACGCTGATTGACGGGCGACAGGTTGTAGAACAGCGGCAGGCGCTCGCTCTCTTTGGTGGTGTAGCGATCTT
O31_rpoB	GAACTTCGAAGCCTGCACGTTCACGGGTCAGACCGCCTGGGCCGAGTGCGGAGATACGACGTTTGTGCGTcATCTCAGACAGCGGGTTGT
O32_nagA	AGGTCCGTGGCTGAATCTGGTAAAAAAAGCACCCATAATCCGAATTTTGTGCGTAAGCCTGATGCCGCGCTGGTCGATTTCCTGTGTGAA
O32_yeiH	TTCCTGCTGGCTTGCTTCCTGGGGCAGAAAGTGTTTGGTCTGGATAAGCACACCAGCTGGTTGtTCGGTGCCGGTAGCAGTATCTGTGGT
O32_ptsP	ACTTTTATACGCTCACCGCTTTTTAACTaCGCGGGTAAATTGACGTCATCTTCCGCCAGACGGCTAAGCTCAATCTCTTCACTAATTAGC
O32_nusA	AATCGTTACATCTGTCATGCTGTTCCTTCCTGCTACAGTTTATTACGCTTCGTCACCGAACCAGCAAATATTACgGGGCAGCCATAATCA
O32_pyrErph	GCGCAAATTCAATAAACTGGCGCTGATATGGTTTCATGCCTTCGCTCCTCATCTTACTTTTCTACAGACAAAAAAAGGCGACTCATCAGT
O33_ydcK	GCTGTCACTTATATATGCACCCTGGCTGATTTCACTATATCGATCCAGACGTTATCCGTTGCGTAAACCTCTCCCCACAGCACGCTGGTG
O33_ptsP	CCACCAGTACACCAAGCAACTGGCGACGTTGAATAATTGGTACGCCTcAAAACGCGCGGAAACGTTCTTCTTTTACGGAGGGGATGTATT
O33_mreB	ACTTGTTTGATGAAGTGCTGGAGCATTTTTTCAGTCACGAAGAAGTCGGaGATAACGCCGTCTTTCATTGGGCGAATGGCAGCAATATTG
O33_cyaA	CTTTCATTATGACGGAAGaGGTTTTCATCAATCAGGAAGAAGCTGACTTCCACACCCAGCGAGGCGGCCCAGTTTTCCAGCAGGCTACAT
**MASC-PCR**	

WT1_mrdB_WT_F	GGGGCCCAGCCTTCATAAACGC
WT1_mrdB_M_F	GGGGGCCCAGCCTTCATAAACGT
WT1_mrdB_R	TGGCATGATGGAGCGTAAAATCGGCC
WT1O31O33_rph_WT_F	CCAGCGCCGCCTTCTGCG
WT1O31O33_rph_M_F	CCAGCGCCGCCTTCTGCC
WT1O31O33_rph_R	CCGCAGTTTCTGTCGGAATTGTGAACG
WT1_rpoB_WT_F	GGCGTGCTGAAGATTGTTAAGGTATATCTGGC
WT1_rpoB_M_F	GGGCGTGCTGAAGATTGTTAAGGTATATCTGGT
WT1_rpoB_R	GACCGATGTTCATACGAGACGGTACGC
WT2_cysE_WT_F	ACCGGTTGCAGCACCACGGA
WT2_cysE_M_F	CCGGTTGCAGCACCACGGC
WT2_cysE_R	CAGCAAAAATTGGTCGCGGTATCATGC
WT2_rpoC_WT_F	ACGTACCGCAGGTGGTAAAGATCTGC
WT2_rpoC_M_F	AGAACGTACCGCAGGTGGTAAAGATCTGT
WT2_rpoC_R	GAAGGAAACGATACCGCTGATTTCAGCCA
WT3_rlmH_WT_F	GAGGGTAAGCGCCGACAGCG

WT3_rlmH_M_F	GGAGGGTAAGCGCCGACAGCT
WT3_rlmH_R	AAACTCTGGAGTTAATGCGTGAAGCTGCA
WT3_ydhZ/pykF_WT_F	GTCTAACAAGTTGTATATTTTTTGAAACGCTGTTTTT
WT3_ydhZ/pykF_M_F	TCTAACAAGTTGTATATTTTTTGAAACGCTGTTTTG
WT3_ydhZ/pykF_R	AGTTTTGCTCATCACGTTGCGCAGATTC
O31_pykF_WT_F	GCTACTCAGGGCGGTAAATCTGCTCG
O31_pykF_M_F	TGCTACTCAGGGCGGTAAATCTGCTCT
O31_pykF_R	CAACTGATGAGCCGTTTTTTCGTTGGTGG
O31_spoT_WT_F	GTATCTGTTTGAAAGCCTGAATCAACTGATTCAAACCT
O31_spoT_M_F	CTGTTTGAAAGCCTGAATCAACTGATTCAAACCG
O31_spoT_R	TATCCTGGTAGGTGGCGGGAGTATCTTCA
O31_wecE_WT_F	CCAAAGAGAGCGAGCGCCTGCT
O31_wecE_M_F	CAAAGAGAGCGAGCGCCTGCC
O31_wecE_R	TACTGGGCAACGTATTTGGTTACACCGTTAAAG
O31_rpoB_WT_F	GACCAGAACAACCCGCTGTCTGAGATT
O31_rpoB_M_F	ACCAGAACAACCCGCTGTCTGAGATG

O31_rpoB_R	GGTCGCGGCTGAACAAGCTGG
O31_gtrB_WT_F	TGAATTCCACCTAAAAACAGTATTG
O31_gtrB_M_F	CTGAATTCCACCTAAAAACAGTATTA
O31_gtrB_R	AACTGATGGACGCCTGAAGC
O32_nagA_WT_F	CTTACGCACAAAATTCGGATTATGGGTGCC
O32_nagA_M_F	GCTTACGCACAAAATTCGGATTATGGGTGCT
O32_nagA_R	GCCCTGGCTCCTTGCTCAGGG
O32_yeiH_WT_F	GTCTGGATAAGCACACCAGCTGGTTGA
O32_yeiH_M_F	GTCTGGATAAGCACACCAGCTGGTTGT
O32_yeiH_R	CAGTGCCATCAGCAGAGGTTTCGC
O32_ptsP_WT_F	AGCATGACTTTTATACGCTCACCGCTTTTTAACTG
O32_ptsP_M_F	GAGCATGACTTTTATACGCTCACCGCTTTTTAACTA
O32_ptsP_R	TCTGGAACGCGAACGACTGACCG
O32_nusA_WT_F	CGTCACCGAACCAGCAAATATTACGGGC
O32_nusA_M_F	CGTCACCGAACCAGCAAATATTACGGGG
O32_nusA_R	AGTGCCAGAAATCGGCGAAGAAGTGA

O32_pyrE/rph_WT_F	GGCGACTGATGAGTCGCCT
O32_pyrE/rph_M_F	GCGACTGATGAGTCGCCC
O32_pyrE/rph_R_F	CATCTACCAGCATTACGCGTC
O33_ydcK_WT_F	CTGTCACTTATATATGCACCCTGGCTGATTTCACTATT
O33_ydcK_M_F	GCTGTCACTTATATATGCACCCTGGCTGATTTCACTATA
O33_ydcK_R	GATCGATCGCGAAACGGTGTTAGCAC
O33_ptsP_WT_F	CTGGCGACGTTGAATAATTGGTACGCCTA
O33_ptsP_M_F	TGGCGACGTTGAATAATTGGTACGCCTC
O33_ptsP_R	CTGTTCGGTCTACCTGGCCGATCATG
O33_mreB_WT_F	GAGCATTTTTTCAGTCACGAAGAAGTCGGC
O33_mreB_M_F	GGAGCATTTTTTCAGTCACGAAGAAGTCGGA
O33_mreB_R	CCCAGCTTTCAGGATTATCCCTTAGTATGTTGAAAAAATTTCG
O33_cyaA_WT_F	GGAAGTCAGCTTCTTCCTGATTGATGAAAACCG
O33_cyaA_M_F	TGGAAGTCAGCTTCTTCCTGATTGATGAAAACCT
O33_cyaA_R	GCCAGCAGACGTGGGTTCGG

Table S5. Summary statistics of DNA and RNA sequencing.

**DNA sample**	**Number of reads after trimming**	**Total nucleotides**	**Mapping coverage in replication rate analysis**
WT	27,408,896	2,751,469,002	591.5
O3	18,485,284	1,849,121,811	398.7
68WT-1	42,885,790	4,304,381,946	927.2
68WT-2	31,031,580	3,113,542,915	670.7
68WT-3	35,851,672	3,596,492,772	774.7
68O3-1	30,502,760	3,060,069,118	660.7
68O3-2	30,277,846	3,037,049,923	655.7
68O3-3	32,031,690	3,214,386,677	694
**RNA sample**	**Number of reads after trimming**	**Total nucleotides**	**Mapping coverage in RNA-Seq**
WT replicate 1	26,809,176	2,410,164,990	404.4
WT replicate 2	31,968,566	3,135,369,779	503.1
O3 replicate 1	27,879,452	2,758,656,031	458.7
O3 replicate 2	31,298,852	3,009,089,965	486.5

68WT-1 replicate 1	26,723,722	2,635,340,009	446
68WT-1 replicate 2	33,294,134	3,254,862,968	529.5
68WT-2 replicate 1	27,074,456	2,667,130,885	445.6
68WT-2 replicate 2	38,831,588	3,845,954,857	580.7
68WT-3 replicate 1	30,401,556	2,964,122,954	485.9
68WT-3 replicate 2	27,790,244	2,746,941,128	453.3
68O3-1 replicate 1	30,092,928	2,986,114,490	490.1
68O3-1 replicate 2	29,176,030	2,863,813,890	497.6
68O3-2 replicate 1	29,930,186	2,913,359,163	481.6
68O3-2 replicate 2	30,194,776	2,975,981,498	489.6
68O3-3 replicate 1	27,111,316	2,654,066,683	472.5
68O3-3 replicate 2	33,374,454	3,297,369,466	519.3

SUPPLEMENTAL FIGURES

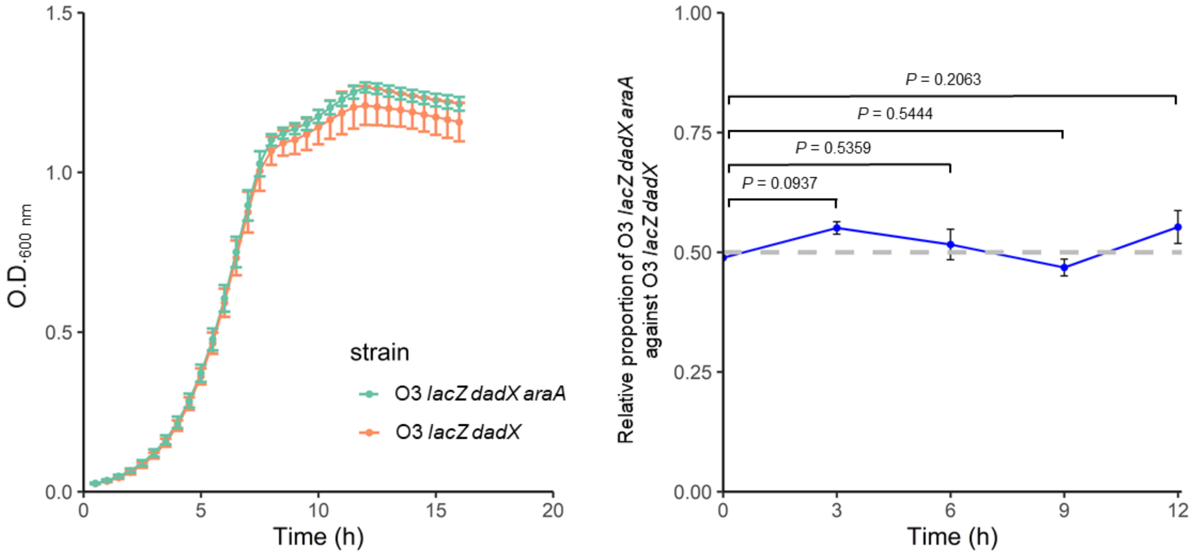

Figure S1. Phenotypic difference between O3 *lacZ dadX* and O3 *lacZ dadX araA* (A) Growth curves of O3 *lacZ dadX* and O3 *lacZ dadX araA* strains. Optical densities were measured every 30 min in 200 μl M9 minimal medium supplemented with 0.4% glucose using a 96-well microplate. (B) Competitive fitness of O3 *lacZ dadX araA* against O3 *lacZ dadX*. O3 *lacZ dadX*, a derivative of *E. coli* MG1655 with two additional replication origins. O3 *lacZ dadX araA*, a derivative of *E. coli* MG1655 with two additional replication origins and arabinose utilization marker.

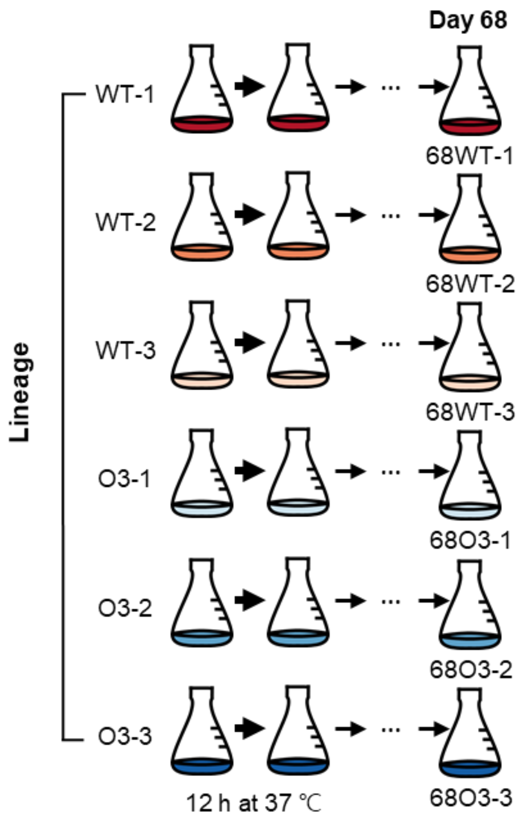

Figure S2. Experimental design of experimental evolution. During the experimental evolution, cells were transferred into a flask with a fresh M9 medium containing 0.4% glucose; each ancestor underwent evolution in three independent flasks (WT-1, WT-2, WT-3; O3-1, O3-2, O3-3). WT, wild-type *E. coli* MG1655; O3, a derivative of *E. coli* MG1655 with two additional replication origins and arabinose utilization marker.

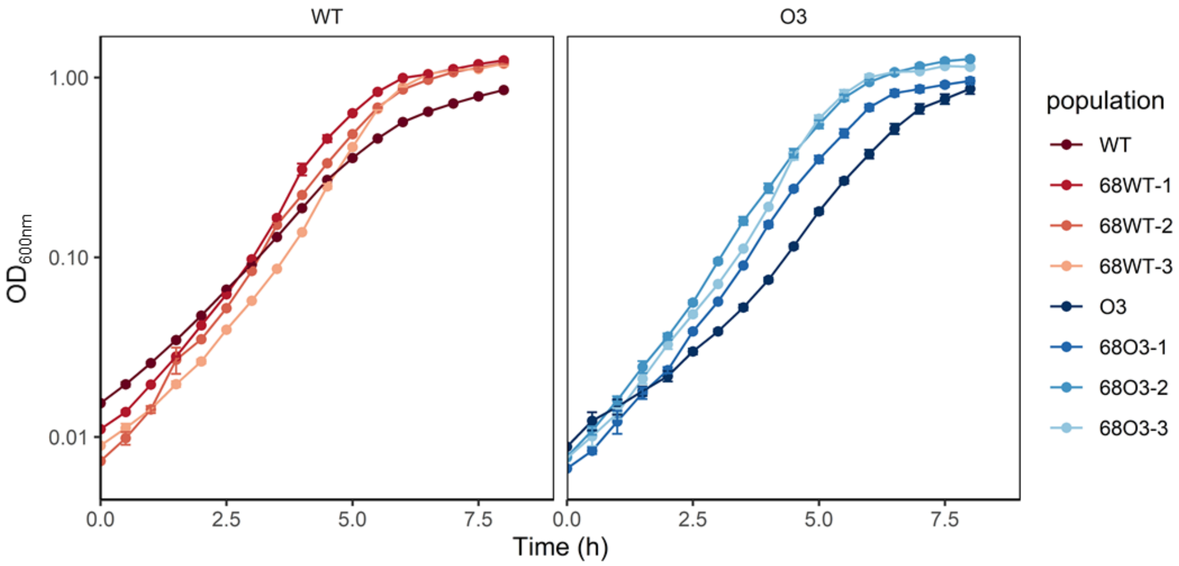

Figure S3. Growth curves of ancestral and evolved populations. Optical densities were measured every 30 min in 200 μl M9 minimal medium supplemented with 0.4% glucose using a 96-well microplate. WT, wild-type *E. coli* MG1655; O3, a derivative of *E. coli* MG1655 with two additional replication origins and arabinose utilization marker.

92

93

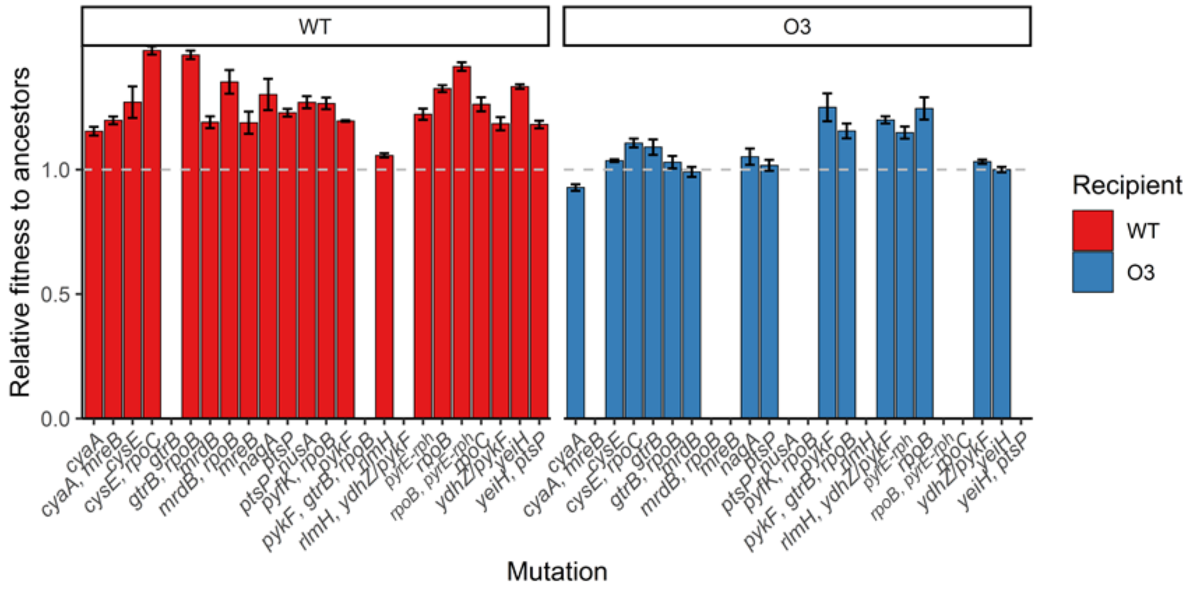

94

95

96 **Figure S4.** Effect of dominant mutations on fitness. Growth rates were measured in M9
97 minimal medium supplemented with 0.4% glucose. Relative fitness was calculated by dividing
98 the growth rates of mutant strains by ancestral strain. Grey line, the relative growth rate of
99 each recipient strain. WT, wild-type *E. coli* MG1655; O3, a derivative of *E. coli* MG1655 with
100 two additional replication origins.

101

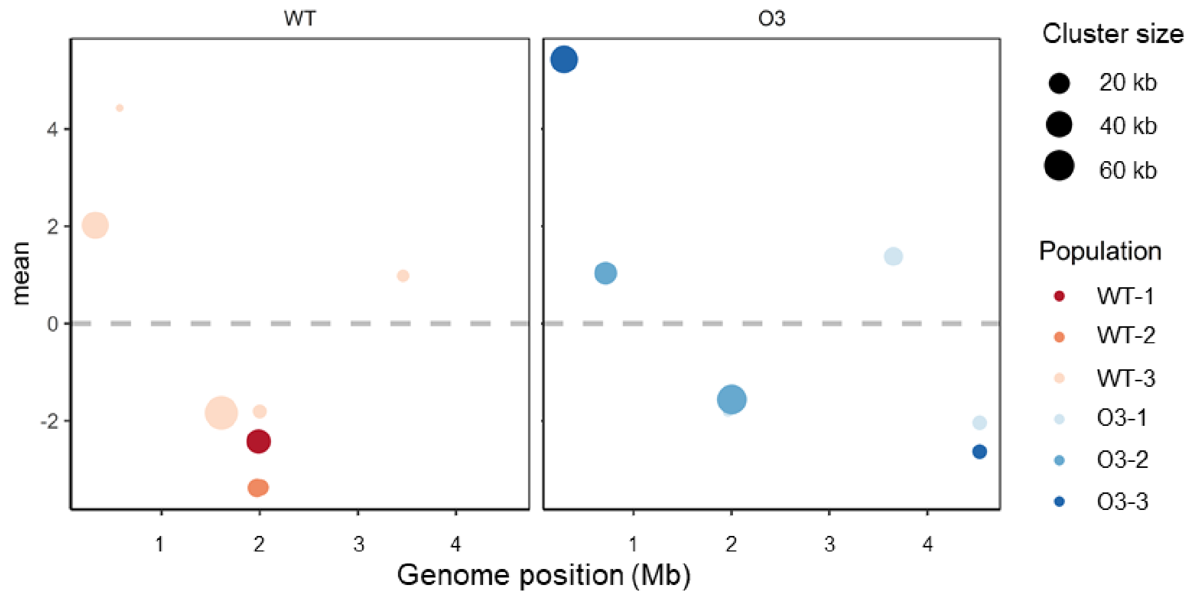

Figure S5. Position-dependent differentially expressed gene (DEG) cluster analysis. DEG cluster analysis was conducted on the final descendant populations (68WT-1 – 68O3-3). DEGs were calculated using the respective ancestral strains as a control. WT, wild-type *E. coli* MG1655; O3, a derivative of *E. coli* MG1655 with two additional replication origins and arabinose utilization marker.

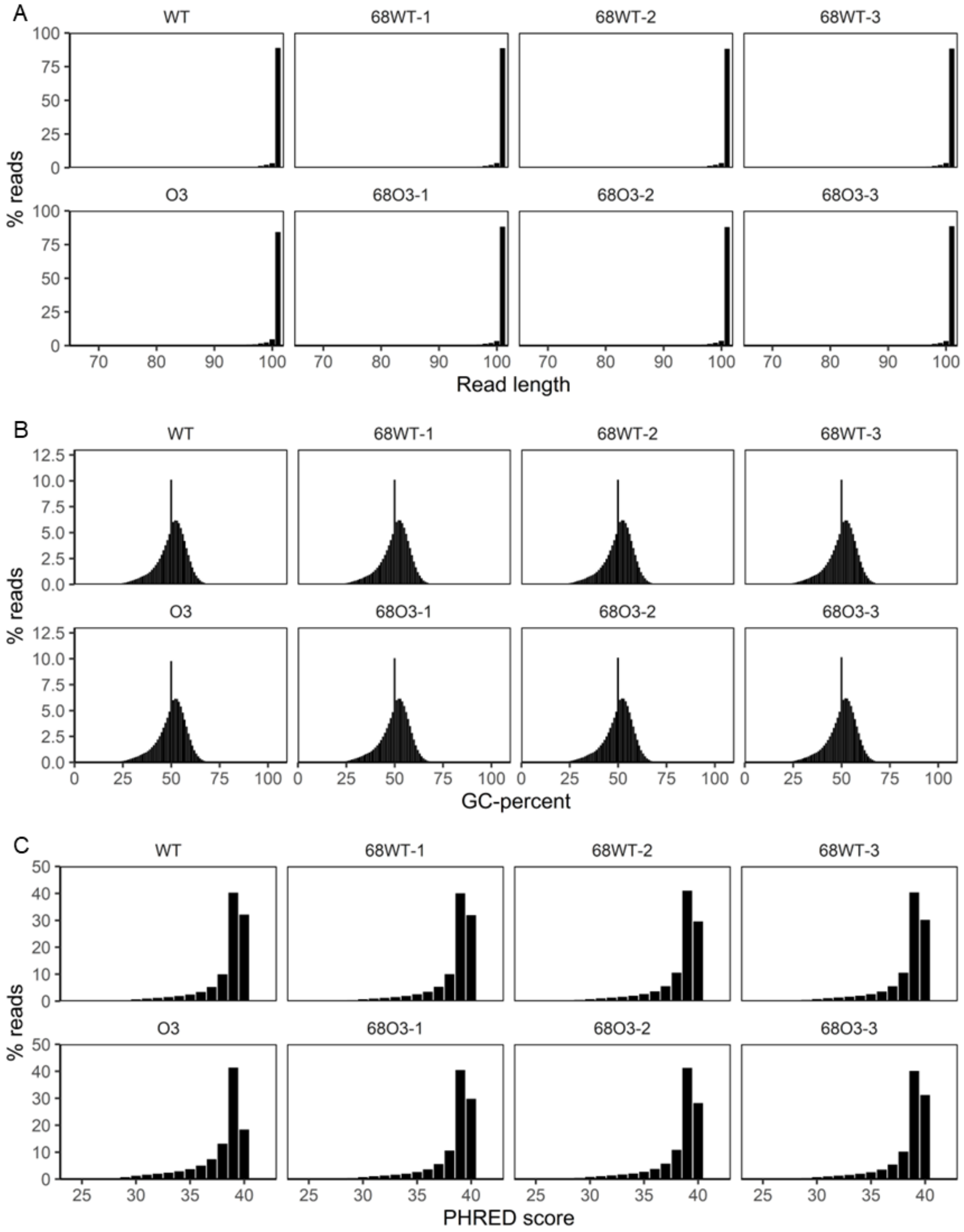

Figure S6. DNA sequencing read quality after read trimming. (A) Length of sequencing reads. (B) GC-contents of sequencing reads. (C) PHRED score of sequencing reads.

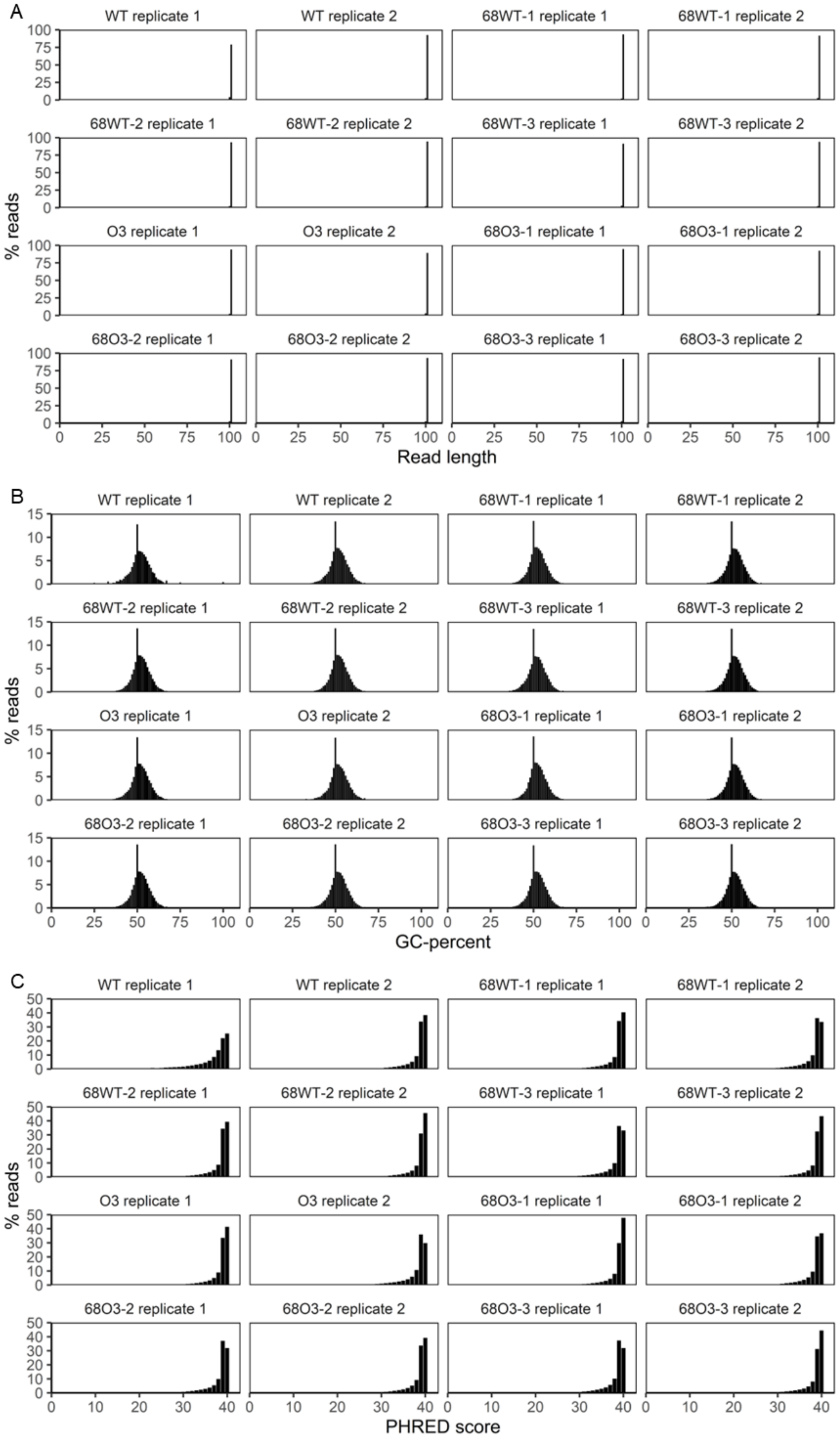
A
WT replicate 1
WT replicate 2
68WT-1 replicate 1
68WT-1 replicate 2
68WT-2 replicate 1
68WT-2 replicate 2
68WT-3 replicate 1
68WT-3 replicate 2
O3 replicate 1
O3 replicate 2
68O3-1 replicate 1
68O3-1 replicate 2
68O3-2 replicate 1
68O3-2 replicate 2
68O3-3 replicate 1
68O3-3 replicate 2
% reads
Read length
B
GC-percent
C
PHRED score

117

Figure S7. RNA sequencing read quality after read trimming. (A) Length of sequencing reads. (B) GC-contents of sequencing reads. (C) PHRED score of sequencing reads.

SUPPLEMENTAL REFERENCES

1. Yang HJ, Kim K, Kwon SK, Kim JF. 2022. *Escherichia coli* cell factories with altered chromosomal replication scenarios exhibit accelerated growth and rapid biomass production. Microb Cell Fact 21:125.
2. Datsenko KA, Wanner BL. 2000. One-step inactivation of chromosomal genes in *Escherichia coli* K-12 using PCR products. Proc Natl Acad Sci U S A 97:6640-5.
3. Edwards RA, Keller LH, Schifferli DM. 1998. Improved allelic exchange vectors and their use to analyze 987P fimbria gene expression. Gene 207:149-57.
4. Lennen RM, Nilsson Wallin AI, Pedersen M, Bonde M, Luo H, Herrgard MJ, Sommer MO. 2016. Transient overexpression of DNA adenine methylase enables efficient and mobile genome engineering with reduced off-target effects. Nucleic Acids Res 44:e36.
